# Antigen-specific immunoglobulin variable region sequencing measures humoral immune response to vaccination in the equine neonate

**DOI:** 10.1371/journal.pone.0177831

**Published:** 2017-05-16

**Authors:** Rebecca L. Tallmadge, Steven C. Miller, Stephen A. Parry, Maria Julia B. Felippe

**Affiliations:** 1 Equine Immunology Laboratory, Department of Clinical Sciences, College of Veterinary Medicine, Cornell University, Ithaca, New York, United States of America; 2 Cornell Statistical Consulting Unit, Cornell University, Ithaca, New York, United States of America; Midwestern University, UNITED STATES

## Abstract

The value of prophylactic neonatal vaccination is challenged by the interference of passively transferred maternal antibodies and immune competence at birth. Taken our previous studies on equine B cell ontogeny, we hypothesized that the equine neonate generates a diverse immunoglobulin repertoire in response to vaccination, independently of circulating maternal antibodies. In this study, equine neonates were vaccinated with 3 doses of keyhole limpet hemocyanin (KLH) or equine influenza vaccine, and humoral immune responses were assessed using antigen-specific serum antibodies and B cell Ig variable region sequencing. An increase (p<0.0001) in serum KLH-specific IgG level was measured between days 21 and days 28, 35 and 42 in vaccinated foals from non-vaccinated mares. In vaccinated foals from vaccinated mares, serum KLH-specific IgG levels tended to increase at day 42 (p = 0.07). In contrast, serum influenza-specific IgG levels rapidly decreased (p≤0.05) in vaccinated foals from vaccinated mares within the study period. Nevertheless, IGHM and IGHG sequences were detected in KLH- and influenza- sorted B cells of vaccinated foals, independently of maternal vaccination status. Immunoglobulin nucleotide germline identity, IGHV gene usage and CDR length of antigen-specific IGHG sequences in B cells of vaccinated foals revealed a diverse immunoglobulin repertoire with isotype switching that was comparable between groups and to vaccinated mares. The low expression of CD27 memory marker in antigen-specific B cells, and of cytokines in peripheral blood mononuclear cells upon *in vitro* immunogen stimulation indicated limited lymphocyte population expansion in response to vaccine during the study period.

## Introduction

Foals present increased susceptibility to sepsis and certain pathogens in comparison to adult horses, a condition often blamed on an incompetent immune system. While the neonatal physiologically differs from the adult immune system for its naïve condition at birth and for described age-dependent changes in antigen presenting cells and T cells, foals can build both cellular and humoral immune responses to pathogens comparably to or with greater values than adult horses [1–5]. In addition, there is growing evidence that the equine fetus and neonate are equipped to respond to antigenic challenge and produce immunoglobulins (Ig) [6–11]. Therefore, vaccination strategies soon after birth could provide timely protection to the foal from infections that cause disease in early age.

The pre-suckle foal is born with endogenous serum IgM and IgG concentrations below protective levels (approximately <30 and <10 mg/dL, respectively) and, in this species, maternal Igs must be passively acquired through the ingestion of colostrum for immediate defense after birth [6,8,12]. Maternal Igs decay with time, generally waning within the first 3 months of age with an IgM half-life of 6–16 days, and IgG half-life of 28–32 days [6,13]. Meanwhile, foals have been shown to produce considerable amounts of endogenous serum IgM and IgG by 2–3 months of age, regardless colostrum ingestion [6–8,14,15].

Earlier studies have illustrated the development of primary and secondary lymphoid tissues in the equine fetus, newborn, and older foal, including the expansion of lymphocyte populations and formation of lymphocyte follicles, and the expression of essential B cell genes and Ig isotypes [8,11,15]. Indeed, experimental and natural infections of mares or equine fetuses have induced antigen-specific IgG responses *in utero* as early as day 200 of gestation [16–18]. In addition, equine fetal B cells at day 100 of gestation already encode low levels of localized Ig mutations and length polymorphisms over the variable, diversity, and joining (VDJ) gene segments; and isotype switching is also detected during fetal life [9–11]. Sequences from pre-suckle neonatal lymph node samples demonstrate an increasing level of Ig diversity throughout gestation, independent of exogenous antigen. Moreover, Ig variable region sequence diversity increases greatly over the initial months of age in response to exogenous antigen [9,10]. Limitations of neonatal Ig repertoire diversity have been reported in other species, either in antigen-specific or unselected contexts, including biased usage of immunoglobulin heavy chain variable (IGHV) genes, absence or limitation of terminal deoxynucleotidyl transferase (TdT) DNA polymerase activity, fewer somatic mutations during B cell affinity maturation, and restricted complementarity-determining region 3 (CDR3) lengths [19–23]. However, similar restrictions have not been found in the equine fetus or neonatal Ig repertoire to date [8–10]. Thus, the equine fetus develops the basic mechanisms necessary to generate humoral responses already in the neonatal period.

Fully mature, functional, and protective immune responses to certain types of vaccines have been reported during the neonatal period in foals, calves, piglets, and fox puppies, suggesting that the use of immunization strategies at this developmental stage has value [24–28]. Indeed, administration of vaccines with adjuvant to neonatal foals as young as 1–6 days old revealed antigen-specific IgG responses [29,30]. In contrast, current vaccination guidelines for horses recommend initiating vaccinations at 3 months of age, and delaying foal vaccination to at least 6 months of age when the dam was vaccinated with the respective vaccine in the last month of gestation; these recommendations aim to overcome a potential maternal antibody interference with the foal’s humoral response (AAEP Vaccination Guidelines Review Task Force, 2015; http://www.aaep.org/custdocs/Foal%20Vaccination%20Chart_8.12.16.pdf). Humoral response to various vaccines used in the young of many domestic species (e.g. cows, pigs, horses, and dogs) were reported to be affected by maternal antibodies [31–36]. The reported suppression was based on the measurement of vaccine antigen-specific serum antibody concentrations in the young through time; however, outcomes vary between types of vaccines and even between studies of the same vaccine, precluding clear conclusions [37–43]. For instance, foal vaccination at 3 months of age has been investigated in many other studies, and serum IgM and IgG responses can be detected after the initial vaccination, in some cases independent of the presence of maternal antibodies [29,44–46].

It is possible that the variation in the outcomes of these studies results partly from the confounding and variable levels of transferred circulating maternal antibodies when measuring endogenous antibody production in the foal, in addition to different IgG half-lives and the antigenic properties of certain vaccine formulations. In order to overcome this limitation, this study describes a novel read-out for humoral vaccine responses using a methodology that measures antigen-specific diversity generated in the IGHV regions expressed on circulating B cells of the foal. In addition, an increase in the diversity of antigen-specific Ig sequences after vaccine boosters corresponds to an increase in the selection for antibodies with greater affinity for the antigen and, consequently, reporting humoral competence.

Taken our previous studies of the immune system of the equine fetus and neonate, we hypothesized that equine neonates generate a diverse antigen-specific Ig response following vaccination, despite the presence of maternal antibodies. To this end, foals received within their first 42 days of age a series of vaccines using a model immunogen (keyhole limpet hemocyanin, KLH) to which horses are naïve or a biologically-relevant commercial vaccine (equine influenza) to which horses are frequently exposed. In addition, their dams were vaccinated or not vaccinated with the same antigens prior to foaling [34,47]. Neonatal humoral responses were evaluated based on antigen-specific serum antibody levels and Ig heavy chain variable region (VDJ) sequence analysis. Altogether, this study measured 1) neonatal antibody production in response to protein vaccines; 2) the diversity of the neonatal antibody repertoire; 3) how the neonatal humoral response changed with vaccine boosters; and 4) how antigen-specific maternally-derived antibodies affected the production and diversity of antibodies in the neonate. A better understanding of neonatal humoral competence may prompt reevaluation of preventative equine neonatal vaccination strategies in order to decrease the period of pathogen susceptibility for young animals and entire herds.

## Materials and methods

### Mares, foals, and experimental design

This study was carried out in strict accordance with the recommendations from Cornell University Center for Animal Resources and Education and Institutional Animal Care and Use Committee for the use of vertebrates in research, under an approved protocol (#2002–0106). All efforts were made to minimize suffering during vaccination and blood collection of foals. Healthy Warmblood and Quarter Horse broodmares carrying pregnancies from Warmblood or Quarter Horse stallions were managed at Cornell University Equine Park, Ithaca, NY on grass pasture during the day, with grass hay and grain supplementation, and observed in a stall overnight in the last month of pregnancy. They received standard vaccinations (tetanus toxoid, Eastern and Western encephalitis, rabies, equine herpesvirus-1) and regular herd deworming based on fecal floatation results. Foals were allowed to suckle colostrum naturally after birth but under observation, and physical examination was performed daily during the study period. Foals with abnormal physical examination, abnormal complete blood cell count, blood IgG concentration values below 800 mg/dL (SNAP® Foal IgG Test, Idexx, Westbrook, MN), or requiring treatment during the 42-day study period were excluded from the study.

**Fig 1:**
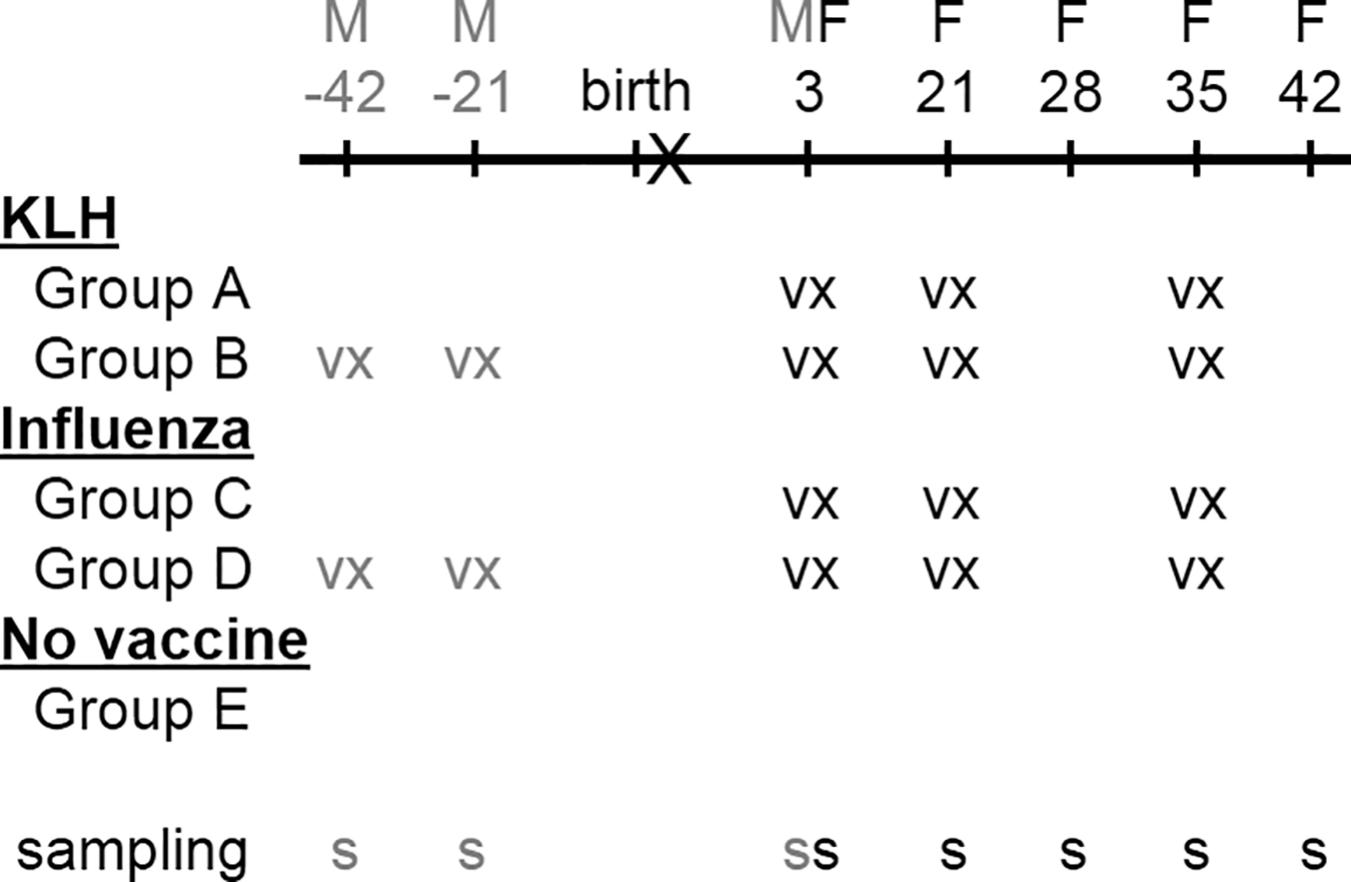
Neonatal vaccination and sampling protocols. Timeline diagram in days describing the vaccination (vx) and sampling (s) schedule for each group. Pregnant mares (M, gray text) were vaccinated on days minus 42 and minus 21, approximately, before foaling with keyhole limpet hemocyanin protein (KLH, Group B) or a commercial influenza vaccine (Group D). Foals (F, black text) were vaccinated on days 3, 21 and 35 of age with KLH protein (Groups A and B) or influenza vaccine (Groups C and D). Blood samples for serum and antigen-specific B cell sorting were collected on days 3, 21, 28, 35 and 42 days of age prior to vaccination. The protocol included a control group (Group E) of non-vaccinated mares and foals with the same sampling times.

Foals were vaccinated intramuscularly on day 3 of age with 2 ml Calvenza™-03 EIV equine influenza killed virus vaccine (Boehringer Ingelheim Vetmedica, Inc., Saint Joseph, MO) or 2 mg KLH in 1 ml saline (Sigma-Aldrich Co., St. Louis, MO) [48]. To determine the effect of boosters on the neonatal humoral response, the same vaccines were repeated on days 21 and 35 (Fig 1). To assess the effect of circulating maternal antibodies acquired through colostrum on the foal’s endogenous antibody production, a group of pregnant mares were vaccinated with 2 intramuscular doses of KLH or influenza vaccine 6 and 3 weeks (42 and 21 days respectively) prior to their foaling date. The study design included 5 groups of foals: Group A) n = 5 foals vaccinated with KLH protein born from non-vaccinated mares during the respective pregnancy; Group B) n = 7 foals vaccinated with KLH protein born from vaccinated mares during the respective pregnancy; Group C) n = 3 foals vaccinated with influenza vaccine born from non-vaccinated mares at least in the last 2 years before the respective pregnancy; Group D) n = 9 foals vaccinated with influenza vaccine born from vaccinated mares during the respective pregnancy; and Group E) n = 4 foals not vaccinated (controls) born from non-vaccinated mares during the respective pregnancy.

Blood samples were collected by jugular venipuncture into vacutainers from mares before vaccination (6 weeks before foaling due date) and on day 3 after foaling (approximately 3 weeks after vaccine booster). Blood samples were collected from foals on days 3, 21, 28, 35, and 42 of age. One foal from Groups A and C could not be sampled on day 42. Complete blood cell counts were performed at birth using the samples collected in EDTA tubes. Peripheral blood mononuclear cells collected in heparin sulfate tubes were isolated using Ficoll gradient (density 1.077, GE Healthcare, Piscataway, NJ) centrifugation at 700 x *g* for 15 min; after cell count and viability measured with Trypan blue (Gibco Life Technologies, Thermo Fisher Scientific, Grand Island, NY) exclusion, cell aliquots (approximately 3 to 5x10^6^ cells) were frozen in 1ml CryoStor CS10 (Stem Cell Technologies, Vancouver, Canada) culture medium, and stored in liquid nitrogen. For serum samples collected in tubes without additives, tubes after blood clotting were centrifuged at 700 x *g* for 15 minutes, and serum aliquots stored at -20^o^ C.

### Serum keyhole limpet hemocyanin (KLH)- or influenza-specific IgG levels

Serum KLH-specific and influenza-specific Ig levels were determined using ELISAs. Briefly, flat-bottom 96-well ELISA plates (Nunc MaxiSorp, Thermo Fisher Scientific) were coated overnight at 4^o^ C with KLH protein (Sigma-Aldrich Co., Saint Louis, MO) diluted at 2 ug/ml in 0.05M carbonate-bicarbonate buffer pH 9.6 (Sigma-Aldrich Co., Saint Louis, MO) [47]. Coated plates were blocked with 50mM Tris pH 8.0 plus 1.0% bovine serum albumin (Sigma-Aldrich Co., Saint Louis, MO) for 30min at room temperature. Two-fold serial dilutions (1:15.6 to 1:2,000) of serum samples from foals and mares of each group were assayed in duplicate; dilutions were performed using 50mM Tris pH 8.0 plus 1.0% bovine serum albumin plus 0.05% Tween 20 solution (Sigma-Aldrich Co., Saint Louis, MO). When needed, serum samples were tested with dilutions from 1:125 to 1:16,000. A goat anti-horse IgG (H&L) peroxidase secondary antibody (1:10,000 dilution, Jackson ImmunoResearch Laboratories, Inc., West Grove, PA) was used to detect KLH-specific Igs bound to the KLH capture antigen. Washing steps between reactions were performed with 50mM Tris pH 8.0 plus 0.05% Tween 20 solution (Sigma-Aldrich Co., Saint Louis, MO). Substrate hydrogen peroxide and chromogen tetramethylbenzidine (Substrate Reagent Pack, R&D Systems, Minneapolis, MN) were added for 30 min, and 1M sulfuric acid (Sigma-Aldrich Co., Saint Louis, MO) was used to stop the reaction. Reaction optical density (O.D.) was immediately determined using a plate photometer (Multiskan EX, Thermo Fischer Scientific, Waltham, MA) at 450nm wavelength, and data captured and analyzed using the equipment Ascent software. After blank subtraction, O.D. values in the linear range were used for analysis; the average O.D. value was determined from duplicate reactions and divided by the serum dilution factor to calculate the normalized O.D.

To determine serum influenza-specific Ig concentrations, a recombinant from baculovirus equine influenza neuraminidase N8 protein with N-terminal histidine tag from influenza virus A/equine/Pennsylvania/1/2007 (H3N8) (NR-13523, BEI Resources, American Type Culture Collection, National Institutes of Health) was used to coat the ELISA plates at 2 ug/ml in carbonate-bicarbonate buffer. Serum serial dilutions from foal (1:15.6 to 1:1,600) and mare (1:800 to 1:6,400) samples were assayed in duplicate as described for the KLH ELISA. A goat anti-horse IgG (H&L) peroxidase secondary antibody (1:20,000 dilution, Jackson ImmunoResearch Laboratories, Inc., West Grove, PA) was used to detect influenza-specific Igs bound to the influenza capture antigen. Results from the influenza ELISA were compared to results obtained from a validated equine influenza virus hemagglutination inhibition assay (EIVHAI) performed by the Cornell University Animal Health Diagnostic Center, Ithaca, NY (Fig A in S1 File).

### Antigen-specific B cell sorting and immunoglobulin transcript amplification

Approximately 5x10^6^ peripheral blood cells in frozen medium as described above from days 21, 28, 35, and 42 from each foal of each group were thawed in 37^o^ C water bath, washed with 10ml RPMI medium plus 10% fetal bovine serum and 1X antimycotic/antibiotic (Gibco Life Technologies, Thermo Fisher Scientific, Grand island, NY), counted and evaluated for cell viability using Trypan blue exclusion. Approximately 2 to 4x10^6^ viable thawed cells were incubated in 500uL RPMI medium with 50uL B cell monoclonal antibody conjugated with allophycocyanin (CD21-APC, clone B-ly4, BD Biosciences, San Jose, CA), 20ug FITC-conjugated KLH (Sigma-Aldrich Co., St. Louis, MO) and 10 ug PE-conjugated equine influenza N8 neuraminidase protein (BEI Resources, NIAID, NIH) [49]. Fluorophore-conjugation kits were used as per manufacturer instruction for 2 mg of KLH (fluorescein, Lynx Rapid Fluorescence Antibody Conjugation Kit, LNK062F, AbDSerotec) or 60 ug influenza neuraminidase (phycoerythrin, Lynx Rapid Fluorescence Antibody Conjugation Kit, LNK022 RPE, AbDSerotec). After 30min incubation at 37^o^ C in a gentle rotator, cells were washed with 10ml phosphate buffered solution (PBS, 37^o^ C), and resuspended in 500uL PBS. Antigen-specific double-positive B cells for CD21/KLH or CD21/influenza were sorted with a BD FACSAria high-speed cell sorter at the Cornell Biomedical Sciences Flow Cytometry Core Lab (Cornell University, Ithaca, NY) (Fig B in S1 File) directly into 300uL RNA lysis buffer (Zymo Research Corporation, Irvine, CA) in individual Eppendorf tubes, and stored at -80^o^ C. Median value for B cells isolated per sample for KLH was 1,147 (range 158, 7,022) and for influenza 2,316 (range 170, 13,213).

RNA from each sample was purified with the Quick-RNA™ MicroPrep as directed (Zymo Research Corporation). IGHM transcripts were amplified with the one-step high-fidelity FideliTaq™ RT-PCR Master Mix (Affymetrix, Santa Clara, CA). The forward primer was conserved among horse IGHV genes (5’ RTGAAGCCCTCRCAGACC 3’) and the reverse primer was in the IGHM heavy chain constant region (5’ CCAAGGAGAAGGTGATGACG 3’); this primer pair yielded amplicons of approximately 450 bases (Fig C in S1 File) [50]. IGHM amplicons were excised from 1% agarose gels and purified with the GeneJET Gel Extraction and DNA Cleanup Micro Kit (ThermoFisher Scientific, Waltham, MA) followed by ligation into the pJET1.2/blunt vector with the CloneJET PCR Cloning Kit (ThermoFisher Scientific, Waltham, MA). Ligations were then transformed into NEB® 5-alpha Competent *E*. *coli* (New England Biolabs, Ipswich, MA). Single colonies were expanded in Luria broth (1% tryptone, 0.5% yeast extract, 0.17M sodium chloride, all from Sigma-Aldrich Co., Saint Louis, MO) with ampicillin (Sigma-Aldrich Co.) and stored as glycerol stocks culture mixed 1:1 with 65% glycerol solution [glycerol (ThermoFisher Scientific, Waltham, MA), 0.1 M magnesium sulfate (Sigma-Aldrich Co.) and 0.025 M Tris-Cl pH 8 (Sigma-Aldrich Co.)]. Frozen glycerol stocks were sent to the Iowa State University DNA Facility (Ames, IA) for plasmid preparation and Sanger sequencing. Twenty-four clones were sequenced per time point for days 21, 28, 35, and 42.

To screen for IGHG expression, FideliTaq™ RT-PCR Master Mix (Affymetrix, Santa Clara, CA) and IGHG constant region primers 5’ GCTCTACTCCCTCAGCAGCA 3’ and 5’ CTACGTTGCAGATGTAGGTC 3’ were used with 0.5 uL RNA (Fig C in S1 File). Resulting amplicons of 71 to 74 bases were visualized on 2% agarose gels.

For antigen-specific IGHG transcript amplification, cDNA was synthesized with Sensiscript Reverse Transcriptase (Qiagen, Valencia CA) from 1 uL RNA. PCR was performed with conserved primers in the 5’ ends of IGHV (5’ GTGGTTCTTCCTCTTTCTGGTG 3’) and IGHG genes (5’ GTCACCATGCTGCTGAGG 3’), and iProof proofreading polymerase (Bio-Rad, Hercules, CA); resulting products of approximately 650 bases in length were excised from agarose gels, cloned and sequenced as described above (Fig C in S1 File).

### B cell immunoglobulin sequence analyses

Sequence content analyses were performed with Geneious version 8.1.8 (http://www.geneious.com) [51]. Replicate sequences were eliminated from the analyses. Germline identity levels were determined by comparing cloned sequences with EquCab2.0 reference genome. Accession numbers KY437157-KY437667 contain Ig sequences determined in this study.

### Antigen-specific memory B cells

Expression of B cell memory markers CD27 and CD38, and B cell reference gene CD79A were measured with quantitative real-time RT-PCR. Briefly, 10 uL reactions were performed in duplicate with iTaq Universal SYBR Green One-Step kit (Bio-Rad Laboratories, Hercules, CA), 1 uL RNA from antigen-specific sorted B lymphocytes, and primers from established assays in a CFX96 real-time PCR detection system (Bio-Rad Laboratories), using the appropriate controls [8,50]. Relative expression was determined with the ΔΔC_T_ (comparative C_T_) method: target gene expression was normalized (subtraction) with the CD79A B cell reference gene expression (ΔC_T_), followed by calibration (subtraction) to target gene expression in antigen-specific cells sorted from a non-vaccinated control foal of the same age (ΔΔC_T_). The fold difference was calculated with the equation 2^-ΔΔCT^, and percent expression was determined by multiplying the fold difference by 100.

### Statistical analyses

Normalized ELISA O.D. values were analyzed using a linear mixed model in R version 3.3.0 (R Foundation for Statistical Computing, Vienna, Austria) using lme4 and lsmeans packages to test for statistically significant differences in serum antigen-specific IgG, and day 3 values were included as covariates in the model to account for individual variation in antibody concentrations; the KLH data values were log transformed to best fit the model, and Tukey’s method was used to adjust p-values for multiple comparisons [52–54]. For all tests, the Type I error was set at 5%. Relationship analyses were performed with Pearson correlation if the data fit a normal distribution or Spearman correlation if the data did not fit a normal distribution, determined by Shapiro-Wilk normality test (GraphPad Prism v6.05 for Windows, San Diego, CA). Data points were plotted with GraphPad.

For Ig sequence data, the Shapiro–Wilk normality test revealed that, in most cases, data did not fit a normal distribution, and the Wilcoxon–Mann–Whitney Rank Sum test with Bonferroni correction for multiple comparisons was used to assess comparisons of nucleotide identity to germline IGHV genes and CDR3 lengths (GraphPad). Chi-square tests were used to measure bias in IGHV gene usage (GraphPad).

## Results

This investigation examined the magnitude (serum antigen-specific IgG levels) and quality (B cell antigen-specific Ig diversity) of the equine neonate humoral immune responses to vaccination in the presence or absence of maternal antibodies.

### Serum keyhole limpet hemocyanin- and influenza-specific IgG levels

**Fig 2:**
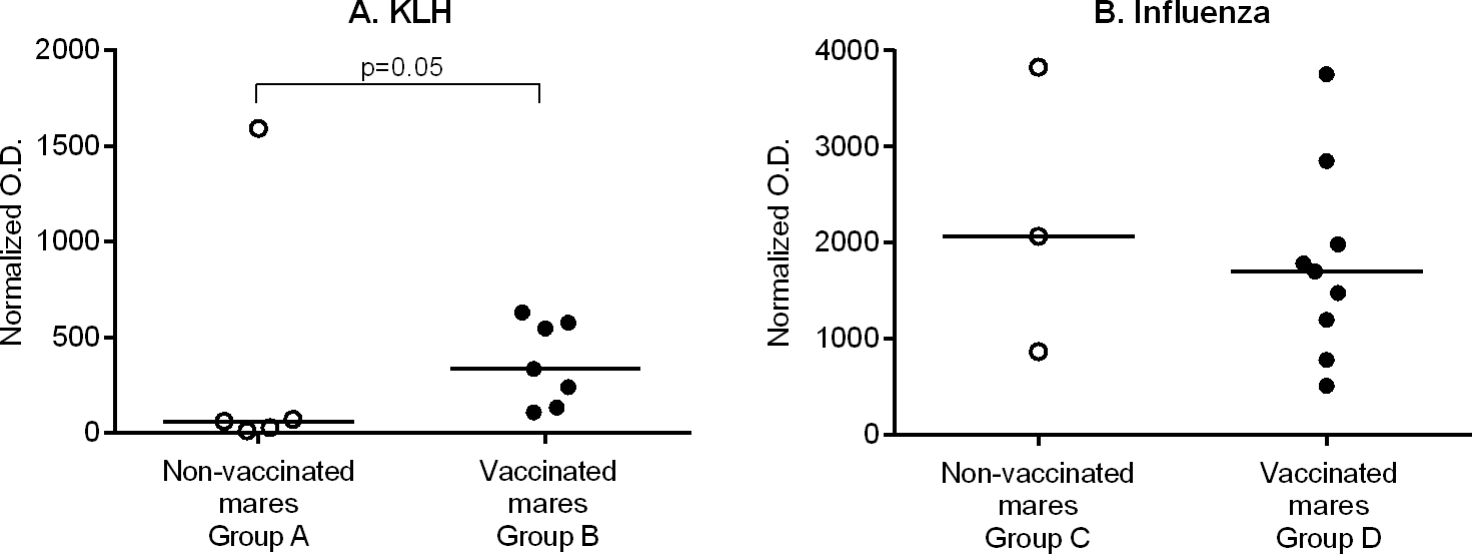
Serum KLH- and influenza-specific IgG levels in vaccinated and non-vaccinated mares. Mares were vaccinated or non-vaccinated at 6 and 3 weeks prior to their expected foaling date, and antigen-specific IgG levels were measured in serum on day 3 after foaling. A) Serum KLH-specific IgG in vaccinated mares (filled circles) trended toward statistically significant greater levels (p = 0.05) in comparison to non-vaccinated mares (open circles). An outlier result is shown in the non-vaccinated group. B) There was no statistical difference (p>0.05) in the serum influenza-specific IgG levels between the vaccinated (filled circles) and non-vaccinated (open circles) mare groups. Note the presence of pre-existing influenza antibodies in the group of non-vaccinated mares despite lack of vaccination with influenza vaccine in the previous 2 years. The horizontal lines indicate median values.

For this study, a group of pregnant mares (n = 7, Group B) was vaccinated with KLH at 6 and 3 weeks prior to their foaling date. A second group of pregnant mares (n = 5, Group A) were not vaccinated. Serum KLH-specific IgG levels on day 3 after foaling in vaccinated mares trended toward statistically greater levels (p = 0.05) in comparison to non-vaccinated mares (Fig 2A). A serum sample from one mare in the non-vaccinated group showed a normalized O.D. value 3 times greater than the highest normalized O.D. in the vaccinated group, and a repeated test confirmed the results, a rare event not published before using this assay; since her foal did not have detectable serum KLH-specific IgG on day 3, the levels measured in the mare serum were likely non-specific to KLH, and the foal was not excluded from the study.

For influenza, mares were vaccinated twice (n = 9, Group D) or not vaccinated (n = 3, Group C) prior to their expected foaling date. Serum influenza-specific IgG levels on day 3 after foaling in vaccinated mares did not differ statistically (p = 0.4) from non-vaccinated mares (Fig 2B). In addition, pre- (day minus 42 before foaling) and post-vaccination (day 3 after foaling) samples were not statistically different (p = 0.2) (Fig A in S1 File).

Blood samples were collected on day 3 of age from foals born from KLH-vaccinated and non-vaccinated mares to measure the passively transferred KLH-specific IgG. A significant (p = 0.01) correlation (Spearman test) for serum KLH-specific IgG levels was observed between the foals’ from all groups and their dams’ sera (r = 0.61). A significant (p = 0.002) Spearman correlation was also identified for influenza-specific IgG levels (r = 0.72).

**Fig 3:**
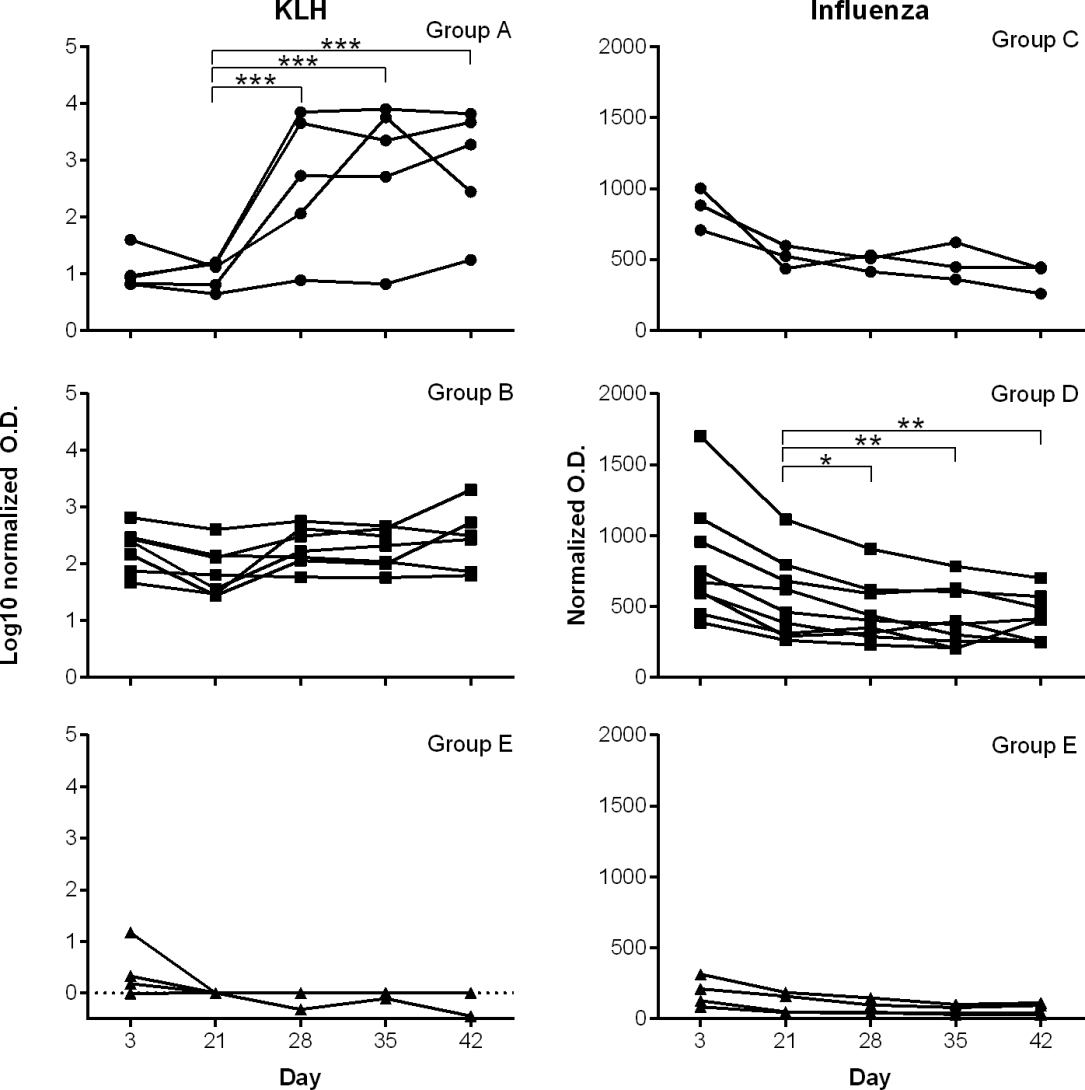
Serum antigen-specific IgG levels in vaccinated and non-vaccinated foals. Serum antigen-specific IgG levels were measured in foals from days 3 to 42 of age. Vaccination was performed on days 3, 21, and 35. Data show mean values of duplicate tests for each foal through time. A statistically significant increase (p<0.0001) in serum KLH-specific IgG was measured between day 21 and days 28, 35 and 42 in KLH-vaccinated foals from non-vaccinated mares (Group A) (log transformed data). In KLH-vaccinated foals from KLH-vaccinated mares (Group B), a trend (p = 0.07) for increased serum KLH-specific IgG levels was observed on day 42 compared to day 21. Passive transfer of maternal antibodies was measured on day 3 in these foals. Non-vaccinated foals from non-vaccinated mares (Group E) had low to undetectable levels of serum KLH-specific IgG (p>0.05). Serum influenza-specific IgG levels tended to decrease (p = 0.09) between days 21 and 42 in influenza-vaccinated foals from non-vaccinated mares (Group C). In influenza-vaccinated foals from influenza-vaccinated mares (Group D), statistically significant decreases in serum influenza-specific IgG levels were detected between days 21 and 28 (p = 0.05), 21 and 35 (p = 0.002), 21 and 42 (p = 0.0003). There were no statistical differences (p>0.05) through time in serum influenza-specific IgG levels in non-vaccinated foals from non-vaccinated mares (Group E). Passive transfer of maternal influenza antibodies was measured on day 3 in all foals, regardless of vaccination status of the dams. Asterisks indicate statistical significance (* p≤0.05, ** p≤0.01, *** p<0.0001).

Serum KLH-specific IgG levels were measured in vaccinated (Group A, from non-vaccinated mares and Group B, from KLH-vaccinated mares) and non-vaccinated (Group E) foals on days 3, 21, 28, 35 and 42 of age. Using the data from day 3 as a covariate to account for individual variation, a statistically significant increase (p<0.0001) in serum KLH-specific IgG levels was measured between day 21 and days 28, 35 and 42 in KLH-vaccinated foals from non-vaccinated mares (Group A) (Fig 3). In KLH-vaccinated foals from KLH-vaccinated mares (Group B), a trend (p = 0.07) for increased serum KLH-specific IgG levels was observed on day 42 compared to day 21 (Fig 3). Non-vaccinated control foals from non-vaccinated mares (Group E) showed low to undetectable levels of serum KLH-specific IgG, which did not change over time (p>0.05) (Fig 3).

Serum influenza-specific IgG levels tended to decrease (p = 0.09) between days 21 and 42 in influenza-vaccinated foals from non-vaccinated mares (Group C) (Fig 3). In influenza-vaccinated foals from influenza-vaccinated mares (Group D), statistically significant decreases in serum influenza-specific IgG levels were detected between days 21 and 28 (p = 0.05), 21 and 35 (p = 0.002), and 21 and 42 (p = 0.0003) (Fig 3). There were no statistical differences (p>0.05) through time in serum influenza-specific IgG levels in non-vaccinated foals from non-vaccinated mares (Group E) (Fig 3). Passive transfer of maternal influenza antibodies was measured on day 3 in all foals, regardless of vaccination status of the dams.

**Fig 4:**
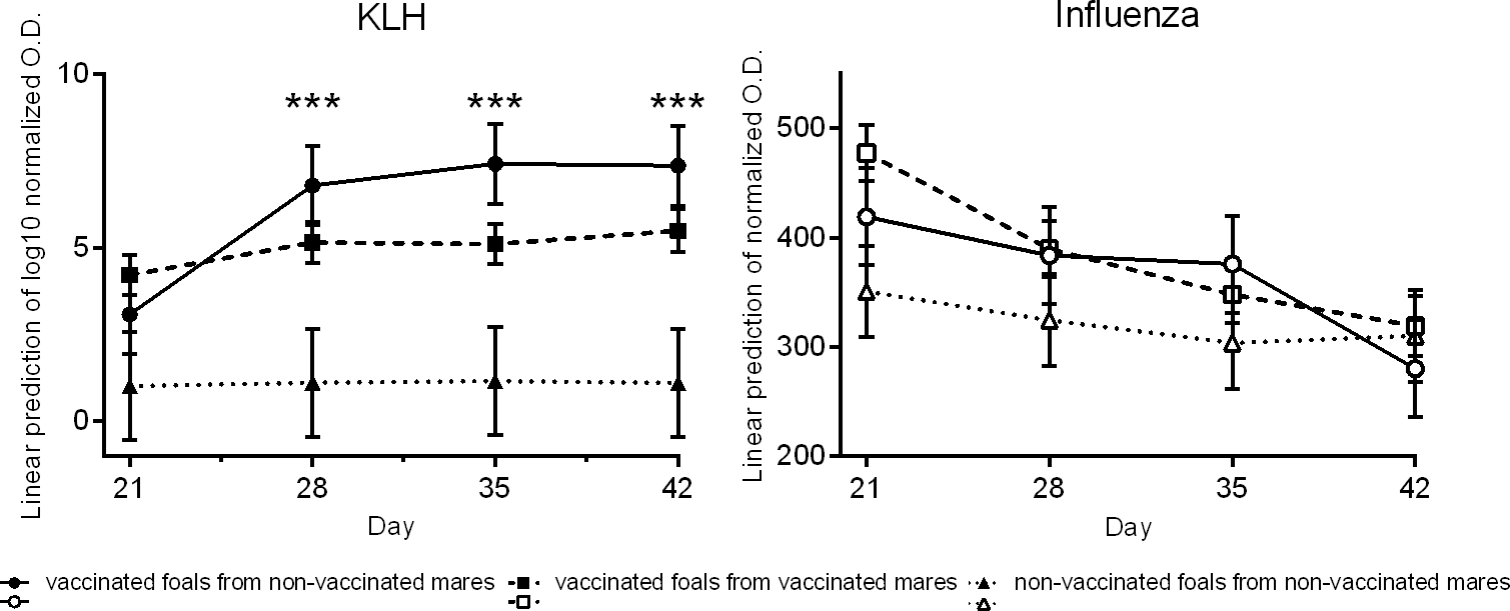
Serum KLH-specific or influenza-specific IgG levels. Linear mixed model analysis was used to compare antigen-specific IgG levels at each time point for the groups: vaccinated foals from non-vaccinated mares (continuous lines; KLH Group A in filled circles; influenza Group C in open circles), vaccinated foals from vaccinated mares (dashed lines; KLH Group B in filled squares; influenza Group D in open squares); non-vaccinated foals from non-vaccinated mares (dotted lines; KLH Group E in filled triangles, influenza Group E in open triangles). Each symbol represents the least square means estimate generated from the data. Significant differences in serum KLH-specific IgG levels were found on days 28, 35, and 42 between Group A and Group E (filled circles versus filled triangles, p<0.0001, denoted by ***). A trend of increased (p = 0.07) serum KLH-specific IgG levels was also found between KLH-vaccinated foals from vaccinated mares (Group B, filled squares) versus non-vaccinated foals from non-vaccinated mares (Group E, filled triangles) on day 42. For influenza, there were no statistical differences (p>0.05) between vaccinated foals (Groups C (open circles) or D (open squares)) and non-vaccinated foals from non-vaccinated mares (Group E, open triangles) at all time points.

A linear mixed model analysis was performed to compare the serum KLH-specific or influenza-specific IgG levels between groups. To account for individual variation in antibody concentrations, day 3 values were included as covariates in the model. Serum KLH-specific IgG levels were not statistically significant different (p≥0.1) between vaccinated foals from non-vaccinated mares (Group A) and vaccinated foals from vaccinated mares (Group B) on days 21, 28, 35, and 42 (Fig 4). Serum KLH-specific IgG levels significantly differed (p≤0.0001) between vaccinated foals from non-vaccinated mares (Group A) and non-vaccinated foals from non-vaccinated mares (Group E) on days 28, 35, and 42. A trend of increased (p = 0.07) serum KLH-specific IgG levels was also found between vaccinated foals from vaccinated mares (Group B) versus non-vaccinated foals from non-vaccinated mares (Group E) on day 42.

For influenza, there were no statistical differences (p>0.05) between influenza-vaccinated foals (Groups C or D) and non-vaccinated foals from non-vaccinated mares (Group E) at all time points (Fig 4).

### Germline identity of antigen-specific IGHM sequences

**Fig 5:**
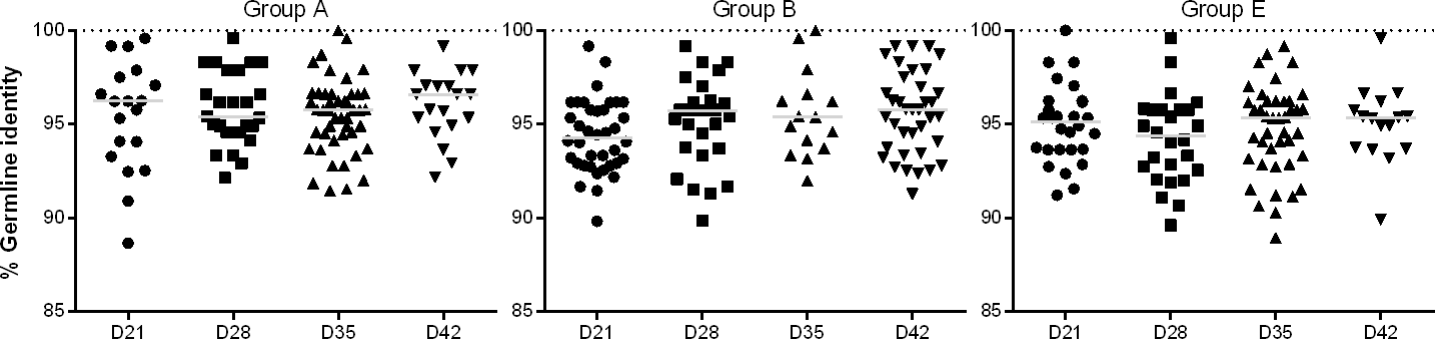
Germline identity of KLH-specific IGHM sequences. The percent of germline nucleotide identity of KLH-specific IGHM sequences for each group is presented for each time point. The value for each cloned PCR product is plotted, and median values are indicated by horizontal lines. No statistically significant differences (p>0.05) were identified over time within a Group or between Groups.

To characterize antigen-specific IgM diversity in response to vaccination, KLH-specific B cells were sorted using flow cytometry from foal peripheral blood samples, and IGHM variable regions were PCR amplified and sequenced from each foal. Overall, IGHM was readily detectable in the KLH-sorted B cells in all foals, regardless of vaccination status. To quantitatively measure the neonatal Ig sequence, the extent of Ig nucleotide diversity from germline (i.e., mutations), IGHV gene usage, and CDR3 lengths were determined for each sequence. KLH-specific IGHM sequences were compared to the equine reference genome EquCab2.0 to determine the extent of diversity from germline present. The median percent of germline nucleotide identity of KLH-specific IGHM sequences ranged from 94.3 to 96.6 across vaccination groups and time points (Fig 5). No statistically significant differences (p>0.05) were identified over time within a group or between groups.

**Fig 6:**
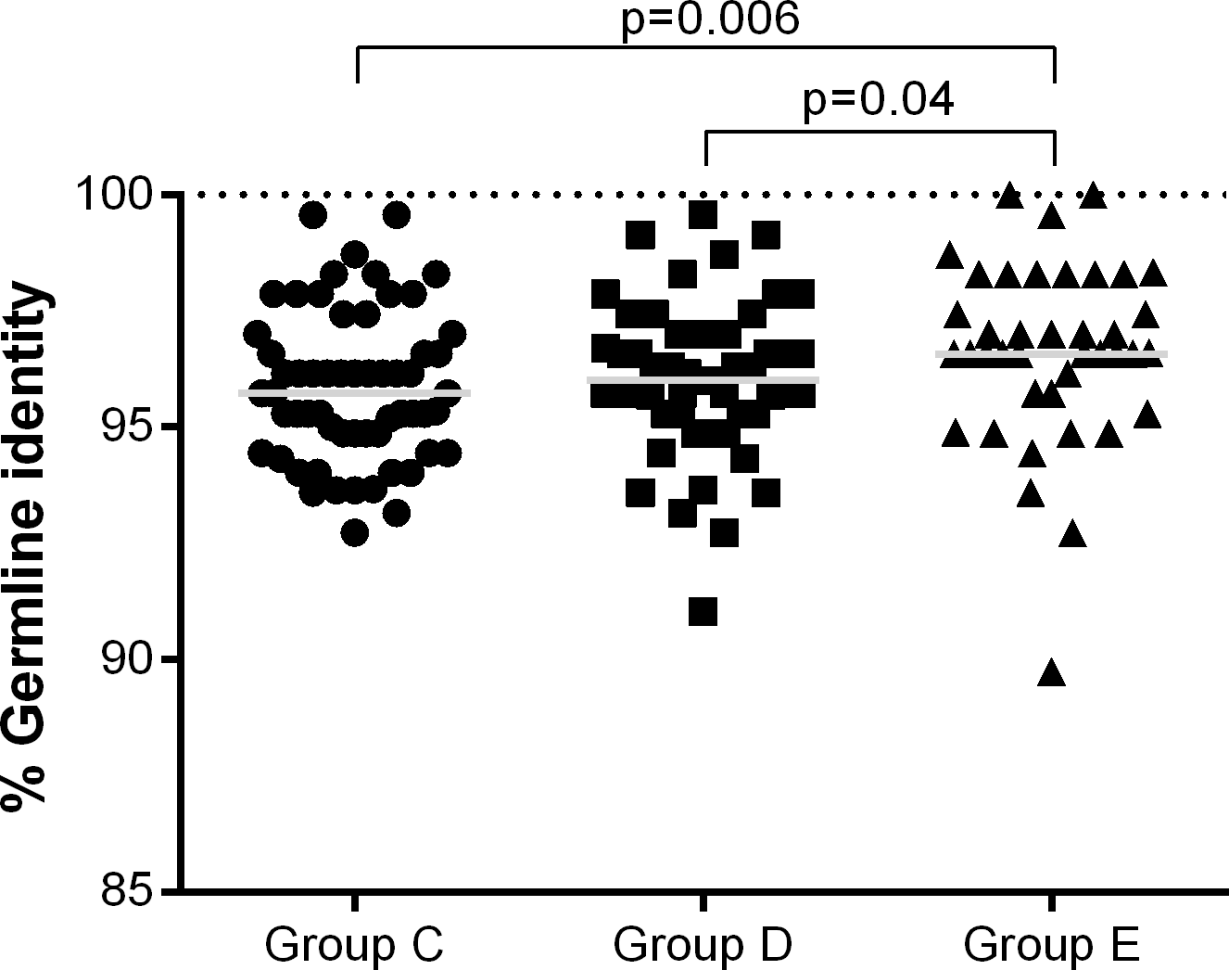
Germline identity of influenza-specific IGHM sequences. The percent of germline nucleotide identity of influenza-specific IGHM sequences for each group is presented for day 42. The value for each cloned PCR product is plotted, and the median values are indicated by horizontal lines. The germline identity values of influenza-specific IGHM transcripts from non-vaccinated foals (Group E) were significantly higher than those from influenza-vaccinated foals (Groups C, p = 0.006, and D, p = 0.04).

Using the same methods, antigen-specific Ig diversity in response to vaccination was measured in sorted influenza-specific B cells from each foal using IGHM variable region PCR amplification, and resulting sequences were compared to the equine reference genome. IGHM transcripts were readily detected on days 21, 28, 35, and 42 in all foals, regardless of vaccination status (Fig 6). Influenza-specific IGHM transcripts were sequenced from day 42 samples and revealed that the germline identity values of influenza-specific IGHM transcripts from non-vaccinated foals (Group E) were significantly higher than vaccinated foals in Groups C (p = 0.006) and D (p = 0.04) (Fig 6). The germline identity values of influenza-specific IGHM transcripts did not differ between vaccinated foal groups C and D (p = 0.3).

### IGHV gene usage in antigen-specific IGHM sequences

To determine how IGHV genes were utilized in the antigen-specific IGHM sequences, the respective IGHV germline gene was identified for each sequence. For KLH-specific IGHM, only 4 different IGHV genes were detected (IGHV2S2, IGHV2S3, IGHV2S4, or IGHV4S1); likewise for influenza-specific IGHM, 4 IGHV genes were detected (IGHV2S2, IGHV2S3, IGHV2S4, or IGHV4S2). The usage of each gene was similar (p>0.05) between the groups (Table 1).

**Table 1:** IGHV gene usage in antigen-specific IGHM sequences.

Vaccine	Group	IGHV2S2	IGHV2S3	IGHV2S4	IGHV4S1	IGHV4S2
KLH	A	20	75	14	1	0
KLH	B	25	74	20	0	0
None*	E	11	81	19	1	0
Influenza	C	5	41	13	0	0
Influenza	D	3	40	8	0	0
None**	E	9	26	4	0	1

* testing for KLH-specific immunoglobulins in non-vaccinated foals.

** testing for influenza-specific immunoglobulins in non-vaccinated foals.

### Antigen-specific IGHM CDR3 length distribution

The CDR3 length distribution of KLH-specific IGHM sequences was also analyzed and no statistical differences (p>0.05) in CDR3 length were found within a group over time or between groups of the same day, (Fig D in S1 File). The CDR3 length distribution of influenza-specific IGHM sequences was compared, and no statistical differences (p>0.05) in CDR3 length were measured (Fig E in S1 File).

### Germline identity of antigen-specific IGHG sequences

To further interrogate the foal Ig response to vaccination, IGHG transcripts were amplified from KLH-sorted B cells on days 28, 35, and 42 from each foal. Initially, conserved primers in the IGHG constant region gene were used in one-step RT-PCR to survey for IGHG mRNA expression of any isotype (Fig C in S1 File). IGHG mRNA was found in most samples from vaccinated foals. IGHG amplicons from mare KLH-specific B cells were rare. Non-vaccinated foals had rare to undetectable IGHG PCR products using this method.

This analysis was then expanded to obtain IGHG transcript sequences, and a conserved IGHV forward primer was paired with a pan-IGHG reverse primer (Fig C in S1 File). These longer IGHV-IGHG RT-PCR products (approximately 600 bases) were consequently more difficult to recover than the small portion of IGHG constant region alone (71 to 74 bases). From vaccinated foal samples, detection of IGHV-IGHG products increased with age, and IGHV-IGHG RT-PCR products were detected in most day 42 KLH-specific B cell samples. Detection of IGHV-IGHG amplicons was variable in mare samples. No IGHV-IGHG sequence could be amplified from non-vaccinated foals (Group E), which validated the specificity of the sorting method. IGHV-IGHG PCR products were cloned and sequenced from day 42 samples, and one to three unique IGHG sequences were obtained per foal despite sampling 24 clones.

**Fig 7:**
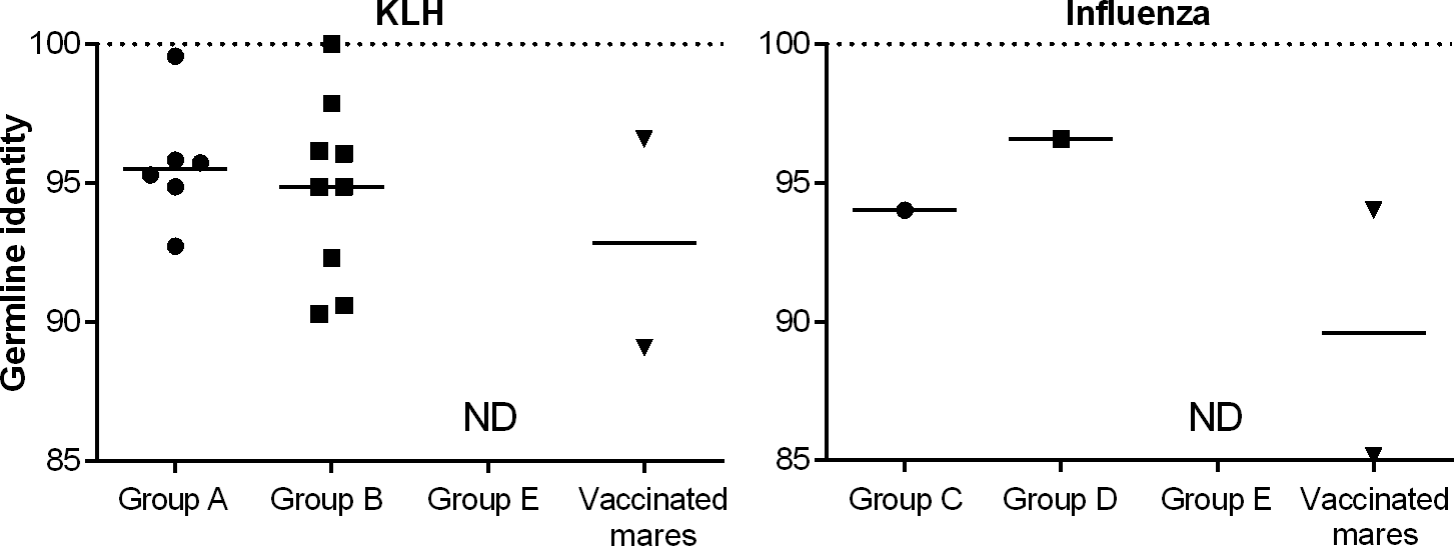
Germline identity of antigen-specific IGHG sequences. The percent of nucleotide germline identity levels of IGHG sequences from KLH-vaccinated foals from non-vaccinated mares (Group A) and KLH-vaccinated foals from KLH-vaccinated mares (Group B) revealed median values of approximately 95%, comparable to that of KLH-specific IGHG sequences sorted from KLH-vaccinated adult mares. Influenza-specific IGHG germline identity levels from influenza-vaccinated foals (Groups C and D) were also near 95%, while those of influenza-vaccinated mares ranged from 85 to 94%. No IGHG sequences could be amplified from non-vaccinated foals (Group E). “ND”, not detected.

For KLH, the median IGHG nucleotide germline identity level was approximately 95% on day 42, comparable (p = 0.7) to values from vaccinated mares (Fig 7).

For influenza, few (1 to 2) unique IGHG sequences were obtained from each group, and statistical analysis was not possible. When no IGHV-IGHG products were detected from foals, additional attempts to amplify antigen-specific IGHV-IGHG transcripts were made with other conserved IGHV forward primers (see supporting information) but these attempts were unsuccessful. Although the forward IGHV primer was designed to be conserved, it is possible that it did not detect some IGHG transcripts.

The use of a pan-IGHG reverse primer allowed amplification of any IgG isotypes, and sequence analysis revealed the use of IGHG1, IGHG3, and IGHG5 isotypes in foals, and IGHG1 and IGHG6 in mares for both KLH- and influenza-specific B cells.

### IGHV gene usage in antigen-specific IGHG sequences

To determine how IGHV genes were utilized in the antigen-specific IGHG sequences, the respective IGHV germline gene was identified for each sequence. For KLH-specific IGHG, 3 different IGHV genes were detected (IGHV2S2, IGHV2S3, IGHV2S4) in vaccinated foal sequences (Table 2), and IGHV2S2 and IGHV2S4 were detected in mare sequences.

**Table 2:** IGHV gene usage in antigen-specific IGHG sequences.

Vaccine	Group	IGHV2S2	IGHV2S3	IGHV2S4
KLH	A	3	1	2
KLH	B foals	1	6	2
KLH	B mares	1	0	1
Influenza	C	0	1	0
Influenza	D foals	0	1	0
Influenza	D mares	0	2	0

Influenza-specific IGHG sequences from vaccinated foals and mares all utilized the IGHV2S3 gene. There were not enough data points for valid chi-square calculations to assess a bias in IGHV usage.

### Antigen-specific IGHG CDR3 length distribution

The measurement of the CDR3 length of KLH-specific IGHG sequences revealed a range of 6 to 24 amino acids in KLH-vaccinated foals (Groups A and B), which spans the range observed in KLH-vaccinated mares (10 to 17) (Table 3). No statistically significant difference (p≥0.3) in IGHG CDR3 length was observed between KLH-vaccinated foal groups (Group A vs. Group B) or between KLH-vaccinated foals and mares. A small and insufficient number of influenza-specific IGHG sequences were obtained for statistical analysis.

**Table 3:** Antigen-specific IGHG CDR3 length distribution.

		CDR3 amino acid length										
Vaccine	Group	6	7	8	10	11	15	16	17	19	21	24
KLH	A			1		1	1	1			2	
KLH	B foals	1	1		1	1	1	1	2			1
KLH	B mares				1				1			
Influenza	C									1		
Influenza	D foals							1				
Influenza	D mares						1			1		

### Antigen-specific memory B cell marker quantification

**Fig 8:**
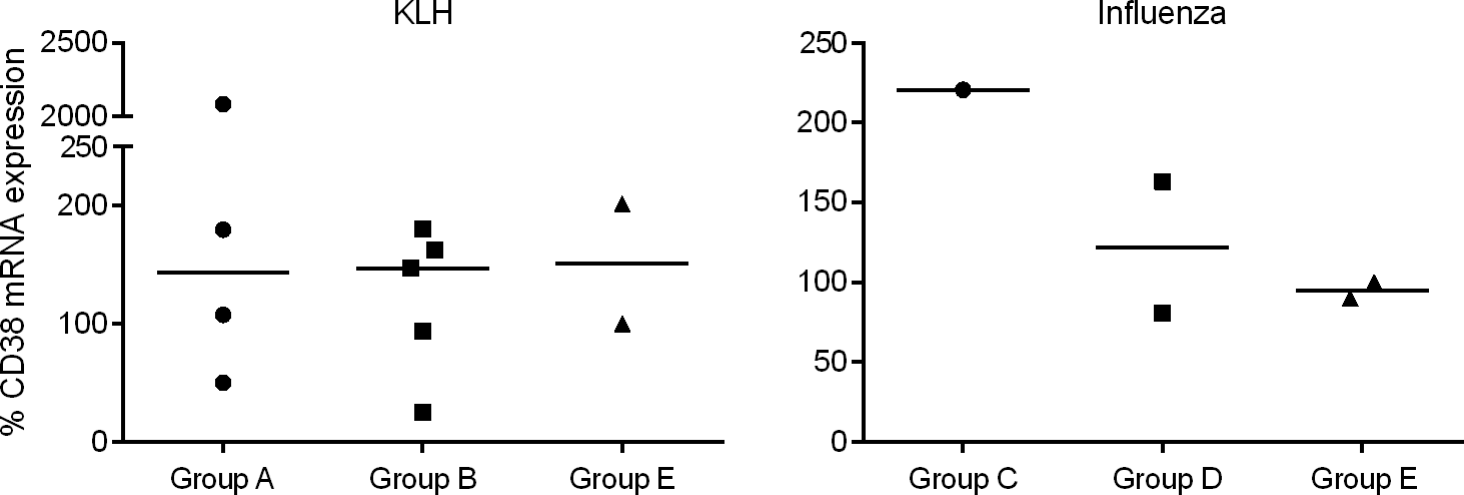
Quantitative CD38 mRNA expression in antigen-specific foal cells on day 42. Relative expression of CD38 memory cell marker is shown by groups: KLH-vaccinated foals from non-vaccinated mares (Group A, n = 5), KLH-vaccinated foals from vaccinated mares (Group B, n = 6), influenza-vaccinated foals from non-vaccinated mares (Group C, n = 3), influenza-vaccinated foals from vaccinated mares (Group D, n = 8), and non-vaccinated foals from non-vaccinated mares (Group E, n = 4). Missing data points indicate lack of detectable CD38 mRNA expression. No statistical difference (p>0.05) in percent CD38 mRNA expression was detected between groups.

As antigen-specific IGHG sequences were not consistently detected until day 42, measurement of memory B cell markers was performed only for day 42 samples. Currently, reagents to detect memory B cell protein markers CD38 and CD27 are lacking for the horse; thus, quantitative RT-PCR was performed. Although KLH-specific B cells from all foals were assayed, CD27 mRNA was detected in only one foal of each vaccination group (Groups A and B), and was not detected in any of the non-vaccinated foals (Group E). CD38 mRNA was detected in 4 of 5 KLH-vaccinated foals from non-vaccinated mares (Group A), in 5 of 6 vaccinated foals from vaccinated mares (Group B), and in 2 of 4 non-vaccinated foals from non-vaccinated mares (Group E) (Fig 8). No statistical difference in percent CD38 mRNA expression was detected between groups.

For influenza-specific B cells, CD27 mRNA was not detected on day 42 samples from Groups C, D, and E. CD38 mRNA was detected in 1 of 3 influenza-vaccinated foals from non-vaccinated mares (Group C), 2 of 8 vaccinated foals from vaccinated mares (Group D), and in 2 of 4 foals from non-vaccinated mares (Group E) (Fig 8). There were insufficient data for statistical analysis of influenza-specific CD38 expression between groups.

### Leukocyte cytokine production after *in vitro* stimulation with KLH or influenza proteins

Peripheral blood mononuclear cells from days 3 and 42 of each foal group were not-stimulated (baseline control) or stimulated *in vitro* with KLH, equine influenza N8 neuraminidase protein or phytohemagglutinin (PHA, positive control). Data analysis revealed no statistical differences (p>0.05) between days 3 and 42, or between groups for the production of IL-4 and IFNγ when cells where stimulated with the immunogens (Fig F in S1 File). Analysis of cytokine production by statistical comparison of IL-4 and IFNγ mean fluorescence intensity (MFI) also resulted in no difference between groups (p>0.05).

## Discussion

This study measured the magnitude and quality of humoral immune responses to vaccination in the equine neonate using antigen-specific serum IgG levels and B cell Ig sequencing. The humoral response to 3 doses of an experimental model protein without adjuvant (KLH) or a commercially available vaccine with proprietary adjuvant (influenza) was measured in foals within their first 42 days of age. In order to test how passively transferred maternal antibodies interfered with the foal humoral immune response, dams were vaccinated or not with the respective immunogens in late gestation. Groups were comprised of vaccinated foals from non-vaccinated mares, vaccinated foals from vaccinated mares using the respective immunogens, and control non-vaccinated foals from non-vaccinated mares.

The dams responded to KLH protein with robust IgG levels, and passive transfer of KLH-specific IgG to the foal through colostrum was successful, validating the use of this experimental model of neonatal vaccination. Because horses are naïve to KLH, which originated from a marine mollusk, it can be used strategically in vaccine studies [47,48]. In contrast, the serum EIVHAI antibody units measured before and after 2 doses of the influenza vaccine did not show a robust vaccine response in the mares based on the absent statistical difference between pre- and post-vaccination samples, and a median 2-fold increase in EIVHAI antibody units after vaccination. In addition, although the mares in Group C experienced a 2-year washout period for influenza vaccination, these mares had influenza-specific serum Ig levels comparable to influenza-vaccinated mares and passive transfer of influenza antibodies through colostrum was detected in their foals (Group C). Thus, pre-existing influenza Igs in Group C mares and poor responses to influenza vaccination by mares in Group D negated our strategy to measure the effect of maternal antibody interference on neonatal endogenous IgG response to influenza vaccination. Overall, this demonstrates that while studying vaccine responses to common horse pathogens (ie, influenza) provides data with the more clinical relevance than responses to a model immunogen like KLH, this approach has analytical limitations when serum Ig levels are the readout. Other studies have shown low antibody response to influenza in adult horses, and possibilities offered were detectable antigen-specific antibodies in the subject horses prior to vaccination (from prior vaccination or natural infection) or variable response to the vaccine among subjects [55,56].

Taken the young age of the foals in this study, the detection of a robust (3 logs greater) KLH-specific IgG production after the first 2 doses of the immunogen 18 days apart was remarkable, and testified to the preparedness of the humoral immune system in the equine neonate, also supported by our and other previous publications that document expression of essential B cell markers and Ig sequence diversity in equine fetal and foal lymphoid tissues [8–11]. The marked amplitude of response was obvious in KLH-vaccinated foals from non-vaccinated mares (Group A); in KLH-vaccinated foals from KLH-vaccinated mares (Group B), 2 foals showed 1 log greater value on day 42 of sampling in comparison to the previous time points, suggesting that the early amplitude of response to KLH was hampered by circulating maternal antibodies, yet not abrogated. Our findings of serum IgG production in response to vaccination are consistent with those of Ryan and Giguere [29], which reported vaccine-specific IgGa and IgG(T) production 3 weeks after vaccination of 3-day-old foals with a commercial cattle vaccine; together, both studies show the earliest Ig response to intramuscular vaccination in the equine neonate. Adequate passive transfer of Ig through colostrum is essential for the survival of the equine neonate, and a valuable strategy against pathogens that challenge the species in early age. Nevertheless, questions remain about the mechanisms by which circulating maternally-derived antibodies affect the development of the neonate’s endogenous humoral immunity, and the overall impact on vaccination response later in life has yet to be defined [57,58]. Other aspects that affect directly and indirectly humoral responses in neonates involve antigen presenting cell competence to activate T cells, co-stimulatory capacity of T cells to stimulate B cells and, importantly, quality of vaccine, including immunogenicity and recognition by different toll-like receptors [59].

Independently of the group, the maternally-derived influenza-specific IgG levels markedly dropped within the first 42 days of age (Groups C and D), at a much faster rate than previously reported [34], which indicates the need to review current influenza vaccination recommendation for foals based on maternal antibody interference criterion. A decreasing serum influenza IgG level within the first 42 days of age in vaccinated foals would also suggest failure of response to influenza vaccination and, taken the contrasting results for KLH response, this finding warrants further investigation on influenza vaccine immunogenicity in the context of the young foal’s immune system. The current AAEP guidelines for influenza vaccination of foals with inactivated vaccine as used in this study call for a 3 dose series between 6 and 12 months of age. Appropriate recommendations for influenza vaccination in human infants has also been a challenge and, to date, there are no influenza vaccines approved for use before 6 months of age; the more recent development of influenza vaccines with greater immunogenicity and the use of novel adjuvants have shown promising results in promoting an immune response in the young [60]. It should be noted that the influenza ELISA capture antigen, a recombinant neuraminidase N8 protein from influenza virus A/equine/Pennsylvania/1/2007 (H3N8), is not from a strain included in the Calvenza™-03 EIV vaccine. However, analysis of 98 EIV N8 protein sequences revealed amino acid identity levels ranging from 88.3 to 100% over the 470 residue protein. Considering these high levels of amino acid identity and the agreement of the influenza ELISA results with the hemaglutination antibody inhibition assay results, it is unlikely that strain differences in the N8 sequence account for the low influenza-specific serum Ig levels reported herein.

Based on previous studies, we expected the challenge of measuring small levels of endogenous serum influenza-specific IgG in the midst of passively-transferred maternal antibodies [33,34]. Therefore, the innovative aspect of our study was the use of B cell Ig variable region sequencing to: first) detect the presence of B cells, known in low numbers, with Ig on the cell surface that recognized the antigen of interest, a measurement not confounded by circulating maternal antibodies; and second) measure the generated B cell Ig diversity and selection after booster doses. Antigen-specific B cells were characterized based on clonality, mutation from DNA germline sequence, heavy chain variable gene usage, complementarity-determining region length, and isotype switching; these are mechanisms used during B cell development in the germinal center of secondary lymphoid tissues to achieve high affinity and specificity for antigens.

IGHM transcripts were readily detectable in the KLH-sorted and influenza-sorted B cells of all foals, regardless of group. This finding suggests that the equine neonate produces *in utero* a repertoire of B cells with Ig variable regions that bind to diverse antigens, including KLH and influenza proteins, and these cells may reflect low-affinity polyreactive natural IgM, perhaps generated by the B-1 cells prevalent in fetal and neonatal periods [61,62]. In addition, a proportion of IgM-expressing B cells are spared from the Ig hypermutation and selection mechanisms performed in lymphoid tissue germinal centers that improve Ig affinity before their isotype switching into IgG [63]. Thus, it was somewhat expected that IGHM transcripts would not markedly differ between vaccinated and non-vaccinated foals through time, and would also be detected in non-vaccinated foals at this early age.

Germline identity indicates how similar or different the generated Ig variable region mRNA sequences compare to DNA sequences, and a lower identity indicates greater Ig diversity through hypermutation, a goal of B cell development during affinity maturation after antigen encounter. For KLH-specific IGHM sequences, germline identity was steady throughout the sampling periods, independently of booster doses. Surprisingly, influenza-specific IGHM sequences showed an increased nucleotide diversity from germline along the IGHV gene region in vaccinated foals when compared to non-vaccinated foals, suggesting a greater emphasis in IGHM mutations using the influenza vaccine product; curiously, other studies have shown that memory IgM B cells harbor more mutations than naïve IgM B cells [64–66]. These results suggest that foals generated, under these conditions, a predominant IgM influenza-specific response to vaccination; however, additional studies are needed to verify this hypothesis. Finally, in agreement, no differences in CDR3 lengths of KLH- or influenza-specific IGHM sequences were found between groups. Whether the carbimmune adjuvant present in the influenza vaccine formulation and not included in the KLH vaccine had a role in promoting Ig diversity of influenza-specific responses is unknown. Adjuvant may skew the T cell response and thereby direct the ultimate humoral and cellular immune responses [67].

KLH- and influenza-specific IGHG sequences were detected, albeit less readily than IGHM sequences, in the vaccinated foals, independently of the vaccination status of their dams, and not in the non-vaccinated foals, which validated the use of Ig sequencing as an approach to detect feeble humoral responses in neonates. IGHG sequences provide a more comprehensive readout of germinal center-generated Ig diversity, with increasing affinity and selection for antigens. As for influenza IGHG, only 1 to 2 unique sequences were obtained from Groups C and D, including the mares, which prevented comparisons of results; nevertheless, the qualitative detection of influenza-specific IGHG sequences testifies to antigenic recognition and B cell development in the vaccinated equine neonate, which was not possible based on serum influenza-specific IgG levels. The low number of unique sequences identified in the foals suggests low frequency of antigen-specific B cells in the circulation during the study period. However, the reason for this same finding in mares is less clear, and possibilities include timing of blood sampling (3 weeks after the booster dose) that resulted in lower number of circulating antigen-specific B cells; or poor immunogenicity of the vaccine, taken the low serum antibody levels post-vaccination. Importantly, the CDR3 length of Ig-specific IGHG sequences in equine neonates overlapped between the vaccinated foal groups, and between vaccinated foals and vaccinated mares, indicating competent accumulation of mutations during the Ig affinity maturation in germinal centers, again independent of vaccination status of the dams. Since the CDR is the variable region of the Ig that provides diversity and specificity after antigen encounter, it has been shown in a vaccine study that the CDR3 amino-acid sequencing is shared among individuals who received the same vaccine, with greater similarities through time, indicating selection and specificity for certain vaccine epitopes [68].

In previous studies, we also demonstrated that general Ig sequence diversity increases from fetal to neonatal period, and between the foal and adult stages of life, without biased usage of IGHV, IGHD and IGHJ gene segments [9]. Likewise in this study, the IGHV gene usage for KLH- or influenza-specific IGHM and IGHG indicated competence for diversity in the equine neonate. For IGHM, IGHV2S3 was the most predominant IGHV gene utilized, followed by IGHV2S2 and IGHV2S4; these IGHV genes are the most commonly used in all stages of equine development, and all 3 genes belong to the equine IGHV subgroup 2 [9,11,69,70]. Usage of IGHV4S1 and IGHV4S2 genes was also identified in several foal antigen-specific B cells, regardless of vaccination status; suggesting that IGHV gene selection is not biased in the response to vaccination in this case. For IGHG transcripts, gene usage of IGHV2S2, IGHV2S3 and IGHV2S4, was identified among vaccinated foals and mares, similar to usage in randomly selected B cells. Equivalent IGHV usage in antigen-specific B cells reported here somewhat differs from the IGHV repertoire of antigen-specific B cells of human infants less than 3 months old, where usage of both overlapping and distinct IGHV genes was documented upon comparison to that of adult antigen-specific B cells [23,71]. More biased IGHV gene usage has been described in fetal mice in comparison to adult mice [19,72–74]. In addition, foal KLH-specific IGHG sequences revealed the use of IGHG1, IGHG3, and IGHG5 isotypes, comparably to the mares’ isotypes and published data in adult horses (reported as IgGa and IgGT), also indicating competence in isotype switching [47]. Influenza-specific IGHG sequences encoded IGHG1 and IGHG6 isotypes, consistent with reports of influenza-specific serum IgGa (encoded by IGHG1) but not IgGT (encoded by IGHG3 and IGHG5) in response to vaccination of adult horses [75].

One other important goal in vaccination of the young is the generation of antigen-specific memory cells. The CD38 molecule is a marker of cell activation, and CD27 a marker of memory [76]. On day 42, after 3 doses of vaccine, the expression of CD27 in antigen-specific B cells was rarely detected in vaccinated foals, whereas the detection of CD38 was more frequently measured, with no statistical differences between groups. The lack of CD27 mRNA detected in foal antigen-specific B cells is consistent with studies of human B cell ontogeny, which revealed that CD27 expression significantly increases by 6 months of age, and the reason for this apparent developmental delay is not clear [77]. Support to this observation was obtained from the low frequency of circulating antigen-specific T cells in the short studied period of 42 days (supporting information). Since other foal vaccination studies have documented cell-mediated immune activation with live vaccines or at later ages, analyses of samples from subsequent time periods (e.g. at 3 months and 6 months of age) would be informative regarding the effect of age and circulating maternal antibodies in immunologic memory development [46,78]. Indeed, physiologic lymphocyte population expansion in response to environmental antigen shows an age-dependent increase that becomes obvious in the foal by 3 months of age, with markedly high peripheral blood lymphocyte counts and hyperplastic lymphoid tissues, including mucosal-associate lymphoid tissues [7,15,79,80]. Therefore, further work is necessary to characterize the timeline of quality and memory development of the immune responses in early life, starting at neonatal phase, not only for horses and domestic animal species, but also for humans [81].

The results presented herein confirm and extend previous studies documenting the equine neonate’s capacity to produce antigen-specific Igs early in life. Despite the fact that primed T cells and memory cells were not numerous in the periphery in the first month of life, the detection of a robust KLH-specific humoral response both at the protein and molecular levels testified for a productive immune activity in secondary lymphoid tissues. In the case of influenza, antigen-specific IGHG sequences detected in vaccinated foals, despite the unmeasured serum response, suggest the presence of immune mechanisms in place that could be explored by different vaccine formulations for an improved amplitude of response. Other studies in the foal have also shown antigen-specific serum Ig levels induced by vaccination in the first week of age, in the absence or presence of maternal antibodies, including different types of vaccine formulations and routes of administration [29,78,82]. Additional vaccine studies of foals 1 to 3 months of age found variable antigen-specific serum Ig responses but observed either protection from clinical disease or evidence of immune priming, underscoring the importance of not relying on antigen-specific serum Ig levels solely as a read-out for vaccine efficacy [46,83].

In conclusion, the data presented herein revealed that vaccination of equine neonates on day 3 of age, followed by booster doses, induced antigen-specific humoral immune responses with a diverse Ig repertoire that undergoes isotype switching measured at the molecular level, independently of circulating maternal antibodies. Importantly, we observed that antigen-specific Ig sequencing provided a detailed measurement of endogenous humoral immune response, and this methodology can be applied to any vaccine or pathogen in any host species. Using this approach, future studies may investigate optimal frequency and interval of boosters for the young horse, effect of vaccination protocols on immunity later in life, and types of vaccines and adjuvants tailored to the young immune system.

## Supporting information

S1 File **Fig A. Antibody level results obtained using influenza ELISA and EIVHAI assay. (A)** Antibody levels from mares in Groups C and D (n = 12) obtained using the influenza ELISA (normalized optical density, O.D.) showed agreement (r = 0.7424, p = 0.0057, Pearson correlation) with the results obtained using EIVHAI (units of antibody). **(B)** Pre- (median 60, minimum 8, maximum 192) and post- (72, 24, 192) vaccination EIVHAI units of antibodies were determined in serum samples from mares from Group D (n = 9) vaccinated with 2 doses of the influenza vaccine. Data points from the same mare before and after vaccination are connected by a line. There was no statistical difference (p = 0.2) between sampling times, and overall median fold-difference between before and after vaccination was 2. **Fig B. Antigen-specific B cell sorting.** Using a high-speed cell sorter, (A) leukocytes with size and granularity characteristic of lymphocytes were initially tested for the expression of the CD21 B cell molecule; (B) from the CD21 positive (CD21^pos^) population, antigen-specific B cells for KLH (KLH^pos^) or influenza (FLU^pos^) were sorted directly into individual tubes containing lysis buffer for subsequent molecular analysis. Note the rare antigen-specific cells in comparison to the overall B cell population. **Fig C. Schematic of primers used for Ig amplification.** A recombined Ig transcript is shown, with the IGHV gene presented in a white box. Complementarity-determining regions (CDRs) 1 and 2 are shown in thin black boxes, CDR3 spans the 3’ end of IGHV gene along with recombined D and J gene regions. The Ig heavy chain constant region is shown in a gray box. 1) To amplify and sequence IGHM transcripts, a conserved primer in the IGHV region was paired with a primer approximately 120 bases into the IGHM constant region, and resulted in products of approximately 450 bases. This primer was designed based on a conserved region among the 522 equine Ig heavy chain sequences available in GenBank (DQ125413-DQ125458, HM175886-HM176092, HQ403608-HQ403643, KC549680-KC549800, KF748698-KF748792, KJ741369-KJ741385), the equine genome sequence IGHV region (NW_001876796), and other projects in our laboratory (50). When no IGHV-IGHG product was detected, other conserved IGHV forward primers (5’ GTGGTTCTTCCTCTTTCTGGTG 3’ and 5’ GCTCCTACATGTGTCCTGTCC 3’) were used singly and in semi-nested PCR but no additional PCR products were obtained. 2) To amplify the IGHG constant region, primers conserved among the 7 IGHG isotypes were used and produced products of 71 to 74 bases. 3) To sequence the variable region of IGHG transcripts, the same conserved primer in the IGHV region used in set 1 was now paired with a reverse primer in the IGHG constant region that was conserved among the 7 IGHG isotypes. IGHV-IGHG products ranged from approximately 550 to 600 bases. **Fig D. KLH-specific IGHM CDR3 length distribution.** The CDR3 amino acid sequence length of KLH-specific IGHM transcripts from foal samples is presented on the x-axis, and the number of unique clones on the y-axis. Data are shown by sample time (age) for each group (Groups A, B and E). **Fig E. Influenza-specific IGHM CDR3 length distribution.** The CDR3 amino acid sequence length of influenza-specific IGHM transcripts from foal samples is presented on the x-axis, and number of unique clones on the y-axis. Data are shown for sample time day 42 for each group (Groups C, D and E). **Fig F. Cytokine production by foal peripheral blood leukocytes stimulated *in vitro* with KLH or influenza protein.** Peripheral blood isolated leukocytes from days 3 (black bars) and 42 (white bars) were not-stimulated (baseline) or stimulated *in vitro* with KLH, influenza or PHA (positive control). After 60hrs in culture, cells were labeled for the expression of IL-4 and IFNg. Percent values of IL-4-positive or IFNg-positive stimulated cells were normalized to (divided by) values measured for the respective non-stimulated samples. Data are shown as relative expression above baseline, and by group: vaccinated foals from non-vaccinated mares (KLH, Group A; influenza, Group C); vaccinated foals from vaccinated mares (KLH, Group B; influenza, Group D); and non-vaccinated foals from non-vaccinated mares (Group E). For PHA-stimulated cells, data are shown for Groups A, B and E. There were no statistical differences (p>0.05) between days 3 and 42, or between groups.
(DOCX)

## References

[1] Breathnach CC, Sturgill-Wright T, Stiltner JL, Adams AA, Lunn DP, Horohov DW. Foals are interferon gamma-deficient at birth. Vet Immunol Immunopathol. 2006 8 15;112(3–4):199–209. doi: 10.1016/j.vetimm.2006.02.010 16621024 10.1016/j.vetimm.2006.02.010

[2] Jacks S, Giguere S, Crawford PC, Castleman WL. Experimental infection of neonatal foals with Rhodococcus equi triggers adult-like gamma interferon induction. Clin Vaccine Immunol CVI. 2007 6;14(6):669–77. doi: 10.1128/CVI.00042-07 17409222 10.1128/CVI.00042-07 PMC1951072

[3] Flaminio MJ, Nydam DV, Marquis H, Matychak MB, Giguere S. Foal monocyte-derived dendritic cells become activated upon Rhodococcus equi infection. Clin Vaccine Immunol CVI. 2009 2;16(2):176–83. doi: 10.1128/CVI.00336-08 19109450 10.1128/CVI.00336-08 PMC2643540

[4] Merant C, Breathnach CC, Kohler K, Rashid C, Meter PV, Horohov DW. Young foal and adult horse monocyte-derived dendritic cells differ by their degree of phenotypic maturity. Vet Immunol Immunopathol. 2009 9 15;131(1–2):1–8. doi: 10.1016/j.vetimm.2009.03.002 19349079 10.1016/j.vetimm.2009.03.002

[5] Sun L, Adams AA, Betancourt A, Stewart JC, Liu C, Horohov DW. The role of proliferation in the regulation of interferon gamma (IFNgamma) expression in foals. Dev Comp Immunol. 2012 3;36(3):534–9. doi: 10.1016/j.dci.2011.09.009 22079897 10.1016/j.dci.2011.09.009

[6] Lavoie JP, Spensley MS, Smith BP, Mihalyi J. Absorption of bovine colostral immunoglobulins G and M in newborn foals. Am J Vet Res. 1989 9;50(9):1598–603. 2508519

[7] Flaminio MJ, Rush BR, Shuman W. Peripheral blood lymphocyte subpopulations and immunoglobulin concentrations in healthy foals and foals with Rhodococcus equi pneumonia. J Vet Intern Med. 1999 6;13(3):206–12. 10357110 10.1892/0891-6640(1999)013<0206:pblsai>2.3.co;2

[8] Tallmadge RL, McLaughlin K, Secor E, Ruano D, Matychak MB, Flaminio MJ. Expression of essential B cell genes and immunoglobulin isotypes suggests active development and gene recombination during equine gestation. Dev Comp Immunol. 2009 9;33(9):1027–38. doi: 10.1016/j.dci.2009.05.002 19442687 10.1016/j.dci.2009.05.002

[9] Tallmadge RL, Tseng CT, King RA, Felippe MJ. Developmental progression of equine immunoglobulin heavy chain variable region diversity. Dev Comp Immunol. 2013 9;41(1):33–43. doi: 10.1016/j.dci.2013.03.020 23567345 10.1016/j.dci.2013.03.020 PMC3672396

[10] Tallmadge RL, Tseng CT, Felippe MJ. Diversity of immunoglobulin lambda light chain gene usage over developmental stages in the horse. Dev Comp Immunol. 2014 10;46(2):171–9. doi: 10.1016/j.dci.2014.04.001 24726757 10.1016/j.dci.2014.04.001 PMC4107094

[11] Battista JM, Tallmadge RL, Stokol T, Felippe MJ. Hematopoiesis in the equine fetal liver suggests immune preparedness. Immunogenetics. 2014 11;66(11):635–49. doi: 10.1007/s00251-014-0799-9 25179685 10.1007/s00251-014-0799-9 PMC4198492

[12] Perryman LE, McGuire TC, Torbeck RL. Ontogeny of lymphocyte function in the equine fetus. Am J Vet Res. 1980 8;41(8):1197–200. 6969560

[13] Sheoran AS, Timoney JF, Holmes MA, Karzenski SS, Crisman MV. Immunoglobulin isotypes in sera and nasal mucosal secretions and their neonatal transfer and distribution in horses. Am J Vet Res. 2000 9;61(9):1099–105. 10976743 10.2460/ajvr.2000.61.1099

[14] Jeffcott LB. The transfer of passive immunity to the foal and its relation to immune status after birth. J Reprod Fertil. 1975 10;(23)(23):727–33. 1060873

[15] Flaminio MJ, Rush BR, Davis EG, Hennessy K, Shuman W, Wilkerson MJ. Characterization of peripheral blood and pulmonary leukocyte function in healthy foals. Vet Immunol Immunopathol. 2000 3 15;73(3–4):267–85. 10713340 10.1016/s0165-2427(00)00149-5

[16] Martin BR, Larson KA. Immune response of equine fetus to coliphage T2. Am J Vet Res. 1973 10;34(10):1363–4. 4748249

[17] Morgan DO, Bryans JT, Mock RE. Immunoglobulins produced by the antigenized equine fetus. J Reprod Fertil. 1975 10;(23)(23):735–8. 1060874

[18] Pusterla N, Conrad PA, Packham AE, Mapes SM, Finno CJ, Gardner IA, et al Endogenous transplacental transmission of Neospora hughesi in naturally infected horses. J Parasitol. 2011 4;97(2):281–5. doi: 10.1645/GE-2657.1 21506870 10.1645/GE-2657.1

[19] Feeney AJ. Lack of N regions in fetal and neonatal mouse immunoglobulin V-D-J junctional sequences. J Exp Med. 1990 11 1;172(5):1377–90. 1700054 10.1084/jem.172.5.1377 PMC2188672

[20] Shiokawa S, Mortari F, Lima JO, Nunez C 3rd FEB, Kirkham PM, et al IgM heavy chain complementarity-determining region 3 diversity is constrained by genetic and somatic mechanisms until two months after birth. J Immunol. 1999 5 15;162(10):6060–70. 10229847

[21] Souto-Carneiro MM, Sims GP, Girschik H, Lee J, Lipsky PE. Developmental changes in the human heavy chain CDR3. J Immunol Baltim Md 1950. 2005 12 1;175(11):7425–36. 10.4049/jimmunol.175.11.7425 16301650

[22] Weitkamp JH, Lafleur BJ, Greenberg HB, JEC Jr. Natural evolution of a human virus-specific antibody gene repertoire by somatic hypermutation requires both hotspot-directed and randomly-directed processes. Hum Immunol. 2005 6;66(6):666–76. doi: 10.1016/j.humimm.2005.02.008 15993712 10.1016/j.humimm.2005.02.008

[23] Williams JV, Weitkamp JH, Blum DL, LaFleur BJ, JEC Jr. The human neonatal B cell response to respiratory syncytial virus uses a biased antibody variable gene repertoire that lacks somatic mutations. Mol Immunol. 2009 12;47(2–3):407–14. doi: 10.1016/j.molimm.2009.08.024 19804909 10.1016/j.molimm.2009.08.024 PMC2788105

[24] Van Drunen Littel-van den Hurk S, Braun RP, Lewis PJ, Karvonen BC, Babiuk LA, Griebel PJ. Immunization of neonates with DNA encoding a bovine herpesvirus glycoprotein is effective in the presence of maternal antibodies. Viral Immunol. 1999;12(1):67–77. doi: 10.1089/vim.1999.12.67 10333244 10.1089/vim.1999.12.67

[25] Blasco E, Lambot M, Barrat J, Cliquet F, Brochier B, Renders C, et al Kinetics of humoral immune response after rabies VR-G oral vaccination of captive fox cubs (Vulpes vulpes) with or without maternally derived antibodies against the vaccine. Vaccine. 2001 9 14;19(32):4805–15. 11535333 10.1016/s0264-410x(01)00211-0

[26] Lopez AM, Hines MT, Palmer GH, Knowles DP, Alperin DC, Hines SA. Analysis of anamnestic immune responses in adult horses and priming in neonates induced by a DNA vaccine expressing the vapA gene of Rhodococcus equi. Vaccine. 2003 9 8;21(25–26):3815–25. 12922115 10.1016/s0264-410x(03)00329-3

[27] Pei Y, Nicholson V, Woods K, Prescott JF. Immunization by intrabronchial administration to 1-week-old foals of an unmarked double gene disruption strain of Rhodococcus equi strain 103+. Vet Microbiol. 2007 11 15;125(1–2):100–10. doi: 10.1016/j.vetmic.2007.05.007 17560744 10.1016/j.vetmic.2007.05.007

[28] Opriessnig T, Patterson AR, Elsener J, Meng XJ, Halbur PG. Influence of maternal antibodies on efficacy of porcine circovirus type 2 (PCV2) vaccination to protect pigs from experimental infection with PCV2. Clin Vaccine Immunol. 2008 3;15(3):397–401. doi: 10.1128/CVI.00416-07 18094109 10.1128/CVI.00416-07 PMC2268265

[29] Ryan C, Giguere S. Equine neonates have attenuated humoral and cell-mediated immune responses to a killed adjuvanted vaccine compared to adult horses. Clin Vaccine Immunol CVI. 2010 12;17(12):1896–902. doi: 10.1128/CVI.00328-10 20943883 10.1128/CVI.00328-10 PMC3008191

[30] Lohmann KL, Lopez AM, Manning ST, Marques FJ, Brownlie R, Allen AL, et al Failure of a VapA/CpG oligodeoxynucleotide vaccine to protect foals against experimental Rhocococcus equi pneumonia despite induction of VapA-specific antibody and interferon-gamma response. Can J Vet Res Rev Can Rech Veterinaire. 2013 7;77(3):161–9. PMC3700440 24101791

[31] Klobasa F, Werhahn E, Butler JE. Regulation of humoral immunity in the piglet by immunoglobulins of maternal origin. Res Vet Sci. 1981 9;31(2):195–206. 7323466

[32] Aghomo HO, Oduye OO, Rupprecht CE. The serological response of young dogs to the Flury LEP strain of rabies virus vaccine. Vet Res Commun. 1990;14(5):415–25. 2247948 10.1007/BF00343220

[33] van Maanen C, Bruin G, Boer-Luijtze E de, Smolders G, Boer GF de. Interference of maternal antibodies with the immune response of foals after vaccination against equine influenza. Vet Q. 1992 1;14(1):13–7. doi: 10.1080/01652176.1992.9694319 1574831 10.1080/01652176.1992.9694319

[34] Wilson WD, Mihalyi JE, Hussey S, Lunn DP. Passive transfer of maternal immunoglobulin isotype antibodies against tetanus and influenza and their effect on the response of foals to vaccination. Equine Vet J. 2001 11;33(7):644–50. 11770984 10.2746/042516401776249435

[35] Endsley JJ, Roth JA, Ridpath J, Neill J. Maternal antibody blocks humoral but not T cell responses to BVDV. Biologicals. 2003 6;31(2):123–5. 12770543 10.1016/s1045-1056(03)00027-7

[36] Nguyen TV, Yuan L, Azevedo MS, Jeong KI, Gonzalez AM, Iosef C, et al High titers of circulating maternal antibodies suppress effector and memory B-cell responses induced by an attenuated rotavirus priming and rotavirus-like particle-immunostimulating complex boosting vaccine regimen. Clin Vaccine Immunol. 2006 4;13(4):475–85. doi: 10.1128/CVI.13.4.475-485.2006 16603615 10.1128/CVI.13.4.475-485.2006 PMC1459641

[37] Burstyn DG, Baraff LJ, Peppler MS, Leake RD, JSG Jr, Manclark CR. Serological response to filamentous hemagglutinin and lymphocytosis-promoting toxin of Bordetella pertussis. Infect Immun. 1983 9;41(3):1150–6. 6309662 10.1128/iai.41.3.1150-1156.1983 PMC264620

[38] Claesson BA, Schneerson R, Robbins JB, Johansson J, Lagergard T, Taranger J, et al Protective levels of serum antibodies stimulated in infants by two injections of Haemophilus influenzae type b capsular polysaccharide-tetanus toxoid conjugate. J Pediatr. 1989 1;114(1):97–100. 2783345 10.1016/s0022-3476(89)80611-0

[39] Daum RS, Siber GR, Ballanco GA, Sood SK. Serum anticapsular antibody response in the first week after immunization of adults and infants with the Haemophilus influenzae type b-Neisseria meningitidis outer membrane protein complex conjugate vaccine. J Infect Dis. 1991 12;164(6):1154–9. 1955715 10.1093/infdis/164.6.1154

[40] Englund JA, Anderson EL, Reed GF, Decker MD, Edwards KM, Pichichero ME, et al The effect of maternal antibody on the serologic response and the incidence of adverse reactions after primary immunization with acellular and whole-cell pertussis vaccines combined with diphtheria and tetanus toxoids. Pediatrics. 1995 9;96(3):580–4. 7659480

[41] Dagan R, Amir J, Mijalovsky A, Kalmanovitch I, Bar-Yochai A, Thoelen S, et al Immunization against hepatitis A in the first year of life: priming despite the presence of maternal antibody. Pediatr Infect J. 2000 11;19(11):1045–52. 10.1097/00006454-200011000-00004 11099084

[42] Kanra G, Yalcin SS, Ceyhan M, Yurdakok K. Clinical trial to evaluate immunogenicity and safety of inactivated hepatitis A vaccination starting at 2-month-old children. Turk J Pediatr. 2000 6;42(2):105–8. 10936974

[43] Nguyen TV, Yuan L, MS PA, Jeong KI, Gonzalez AM, Iosef C, et al Low titer maternal antibodies can both enhance and suppress B cell responses to a combined live attenuated human rotavirus and VLP-ISCOM vaccine. Vaccine. 2006 3 20;24(13):2302–16. doi: 10.1016/j.vaccine.2005.11.043 16361002 10.1016/j.vaccine.2005.11.043

[44] Minke JM, Toulemonde CE, Dinic S, Cozette V, Cullinane A, Audonnet JC. Effective priming of foals born to immune dams against influenza by a canarypox-vectored recombinant influenza H3N8 vaccine. J Comp Pathol. 2007 7;137 Suppl 1:S76–80. 17559865 10.1016/j.jcpa.2007.04.016

[45] Sturgill TL, Horohov DW. Vaccination Response of Young Foals to Keyhole Limpet Hemocyanin: Evidence of Effective Priming in the Presence of Maternal Antibodies. J Equine Vet Sci. 2010;30:359–64.

[46] Davis EG, Bello NM, Bryan AJ, Hankins K, Wilkerson M. Characterisation of immune responses in healthy foals when a multivalent vaccine protocol was initiated at age 90 or 180 days. Equine Vet J. 2015 11;47(6):667–74. doi: 10.1111/evj.12350 25205445 10.1111/evj.12350

[47] Ainsworth DM, Appleton JA, Antczak DF, Santiago MA, Aviza G. IgG antibody responses to an inhaled antigen in horses with “heaves” (recurrent airway obstruction). Vet Immunol Immunopathol. 2002 1 15;84(3–4):169–80. 11777532 10.1016/s0165-2427(01)00400-7

[48] Edmonds JD, Horohov DW, Chapmat MR, Pourciau SS, Antoku K, Snedden K, et al Altered immune responses to a heterologous protein in ponies with heavy gastrointestinal parasite burdens. Equine Vet J. 2001 11;33(7):658–63. 11770986 10.2746/042516401776249282

[49] Ibrahim S, Steinbach F. Immunoprecipitation of equine CD molecules using anti-human MABs previously analyzed by flow cytometry and immunohistochemistry. Vet Immunol Immunopathol. 2012 1 15;145(1–2):7–13. doi: 10.1016/j.vetimm.2011.07.021 22070824 10.1016/j.vetimm.2011.07.021

[50] Badial PR, Tallmadge RL, Miller S, Stokol T, Richards K, Borges AS, et al Applied Protein and Molecular Techniques for Characterization of B Cell Neoplasms in Horses. Clin Vaccine Immunol CVI. 2015 11;22(11):1133–45. doi: 10.1128/CVI.00374-15 26311245 10.1128/CVI.00374-15 PMC4622111

[51] Kearse M, Moir R, Wilson A, Stones-Havas S, Cheung M, Sturrock S, et al Geneious Basic: an integrated and extendable desktop software platform for the organization and analysis of sequence data. Bioinforma Oxf Engl. 2012 6 15;28(12):1647–9. 10.1093/bioinformatics/bts199 PMC3371832 22543367

[52] R Core Team. R: A language and environment for statistical computing. [Internet]. Vienna, Austria: R Foundation for Statistical Computing; 2016 Available from: https://www.R-project.org/

[53] Bates D, Maechler M, Bolker B, Walker S. Fitting Linear Mixed-Effects Models Using lme4. J Stat Softw. 2015;67(1):1–48.

[54] Lenth R. Least-Squares Means: The R Package lsmeans. J Stat Softw. 2016;69(1):1–33.

[55] Mumford EL, Traub-Dargatz JL, Carman J, Callan RJ, Collins JK, Goltz KL, et al Occurrence of infectious upper respiratory tract disease and response to vaccination in horses on six sentinel premises in northern Colorado. Equine Vet J. 2003 1;35(1):72–7. 12553466 10.2746/042516403775467379

[56] Holmes MA, Townsend HG, Kohler AK, Hussey S, Breathnach C, Barnett C, et al Immune responses to commercial equine vaccines against equine herpesvirus-1, equine influenza virus, eastern equine encephalomyelitis, and tetanus. Vet Immunol Immunopathol. 2006 5 15;111(1–2):67–80. doi: 10.1016/j.vetimm.2006.01.010 16476488 10.1016/j.vetimm.2006.01.010

[57] Clarke E, Kampmann B, Goldblatt D. Maternal and neonatal pneumococcal vaccination—where are we now? Expert Rev Vaccines. 2016 10;15(10):1305–17. doi: 10.1586/14760584.2016.1167602 26998805 10.1586/14760584.2016.1167602

[58] Mwila K, Chilengi R, Simuyandi M, Permar SR, Becker-Dreps S. Contribution of Maternal Immunity to Decreased Rotavirus Vaccine Performance in Low- and Middle-Income Countries. Clin Vaccine Immunol CVI. 2017 1;24(1):e00405–16. doi: 10.1128/CVI.00405-16 27847365 10.1128/CVI.00405-16 PMC5216432

[59] Willems F, Vollstedt S, Suter M. Phenotype and function of neonatal DC. Eur J Immunol. 2009 1;39(1):26–35. doi: 10.1002/eji.200838391 19137537 10.1002/eji.200838391

[60] Holbrook BC, D’Agostino RBJ, Parks GD, Alexander-Miller MA. Adjuvanting an inactivated influenza vaccine with flagellin improves the function and quantity of the long-term antibody response in a nonhuman primate neonate model. Vaccine. 2016 9 7;34(39):4712–7. doi: 10.1016/j.vaccine.2016.08.010 27516064 10.1016/j.vaccine.2016.08.010 PMC5017597

[61] Wang H, Coligan JE, Morse HC 3rd. Emerging Functions of Natural IgM and Its Fc Receptor FCMR in Immune Homeostasis. Front Immunol. 2016;7:99 doi: 10.3389/fimmu.2016.00099 27014278 10.3389/fimmu.2016.00099 PMC4791374

[62] Prieto JB, Tallmadge RL, Felippe MJB. Developmental expression of B cell molecules in equine lymphoid tissues. Vet Immunol Immunopathol. 2017;183:60–71. doi: 10.1016/j.vetimm.2016.12.004 28063478 10.1016/j.vetimm.2016.12.004 PMC5267323

[63] Eisen HN. Affinity enhancement of antibodies: how low-affinity antibodies produced early in immune responses are followed by high-affinity antibodies later and in memory. Cancer Immunol Res. 2014 5;2(5):381–92. doi: 10.1158/2326-6066.CIR-14-0029 24795350 10.1158/2326-6066.CIR-14-0029

[64] Galson JD, Clutterbuck EA, Truck J, Ramasamy MN, Munz M, Fowler A, et al BCR repertoire sequencing: different patterns of B-cell activation after two Meningococcal vaccines. Immunol Cell Biol. 2015 11;93(10):885–95. doi: 10.1038/icb.2015.57 25976772 10.1038/icb.2015.57 PMC4551417

[65] Krishnamurty AT, Thouvenel CD, Portugal S, Keitany GJ, Kim KS, Holder A, et al Somatically Hypermutated Plasmodium-Specific IgM(+) Memory B Cells Are Rapid, Plastic, Early Responders upon Malaria Rechallenge. Immunity. 2016 8 16;45(2):402–14. doi: 10.1016/j.immuni.2016.06.014 27473412 10.1016/j.immuni.2016.06.014 PMC5118370

[66] Hara Y, Tashiro Y, Murakami A, Nishimura M, Shimizu T, Kubo M, et al High affinity IgM(+) memory B cells are generated through a germinal center-dependent pathway. Mol Immunol. 2015 12;68(2 Pt C):617–27. 26514429 10.1016/j.molimm.2015.10.003

[67] Bergmann-Leitner ES, Leitner WW. Adjuvants in the Driver’s Seat: How Magnitude, Type, Fine Specificity and Longevity of Immune Responses Are Driven by Distinct Classes of Immune Potentiators. Vaccines. 2014 4 10;2(2):252–96. doi: 10.3390/vaccines2020252 26344620 10.3390/vaccines2020252 PMC4494256

[68] Galson JD, Truck J, Fowler A, Clutterbuck EA, Munz M, Cerundolo V, et al Analysis of B Cell Repertoire Dynamics Following Hepatitis B Vaccination in Humans, and Enrichment of Vaccine-specific Antibody Sequences. EBioMedicine. 2015 12;2(12):2070–9. doi: 10.1016/j.ebiom.2015.11.034 26844287 10.1016/j.ebiom.2015.11.034 PMC4703725

[69] Sun Y, Wang C, Wang Y, Zhang T, Ren L, Hu X, et al A comprehensive analysis of germline and expressed immunoglobulin repertoire in the horse. Dev Comp Immunol. 2010 9;34(9):1009–20. doi: 10.1016/j.dci.2010.05.003 20466019 10.1016/j.dci.2010.05.003

[70] Walther S, Rusitzka TV, Diesterbeck US, Czerny C-P. Equine immunoglobulins and organization of immunoglobulin genes. Dev Comp Immunol. 2015 12;53(2):303–19. doi: 10.1016/j.dci.2015.07.017 26219564 10.1016/j.dci.2015.07.017

[71] Prabakaran P, Chen W, Singarayan MG, Stewart CC, Streaker E, Feng Y, et al Expressed antibody repertoires in human cord blood cells: 454 sequencing and IMGT/HighV-QUEST analysis of germline gene usage, junctional diversity, and somatic mutations. Immunogenetics. 2012 5;64(5):337–50. doi: 10.1007/s00251-011-0595-8 22200891 10.1007/s00251-011-0595-8 PMC6953429

[72] Perlmutter RM, Kearney JF, Chang SP, Hood LE. Developmentally controlled expression of immunoglobulin VH genes. Science. 1985 3 29;227(4694):1597–601. 3975629 10.1126/science.3975629

[73] Yancopoulos GD, Desiderio SV, Paskind M, Kearney JF, Baltimore D, Alt FW. Preferential utilization of the most JH-proximal VH gene segments in pre-B-cell lines. Nature. 1984 10 25;311(5988):727–33. 6092962 10.1038/311727a0

[74] Jeong HD, Teale JM. Comparison of the fetal and adult functional B cell repertoires by analysis of VH gene family expression. J Exp Med. 1988 8 1;168(2):589–603. 3261774 10.1084/jem.168.2.589 PMC2189009

[75] Muirhead TL, McClure JT, Wichtel JJ, Stryhn H, Frederick Markham RJ, McFarlane D, et al The effect of age on serum antibody titers after rabies and influenza vaccination in healthy horses. J Vet Intern Med. 2008 6;22(3):654–61. doi: 10.1111/j.1939-1676.2008.0091.x 18466246 10.1111/j.1939-1676.2008.0091.x

[76] Agematsu K. [Molecules involved in characteristics of naive/memory B cells]. Nihon Rinsho Meneki Gakkai Kaishi. 2004 10;27(5):309–14. 15559319 10.2177/jsci.27.309

[77] Avanzini MA, Maccario R, Belloni C, Carrera G, Bertaina A, Cagliuso M, et al B lymphocyte subsets and their functional activity in the early months of life. Int J Immunopathol Pharmacol. 2010 3;23(1):247–54. doi: 10.1177/039463201002300122 20378010 10.1177/039463201002300122

[78] Bordin AI, Pillai SD, Brake C, Bagley KB, Bourquin JR, Coleman M, et al Immunogenicity of an electron beam inactivated Rhodococcus equi vaccine in neonatal foals. PloS One. 2014;9(8):e105367 doi: 10.1371/journal.pone.0105367 25153708 10.1371/journal.pone.0105367 PMC4143214

[79] Zink MC, Johnson JA. Cellular constituents of clinically normal foal bronchoalveolar lavage fluid during postnatal maturation. Am J Vet Res. 1984 5;45(5):893–7. 6732021

[80] Banks EM, Kyriakidou M, Little S, Hamblin AS. Epithelial lymphocyte and macrophage distribution in the adult and fetal equine lung. J Comp Pathol. 1999 1;120(1):1–13. 10098012 10.1053/jcpa.1998.0250

[81] Pichichero ME. Challenges in vaccination of neonates, infants and young children. Vaccine. 2014 6 30;32(31):3886–94. doi: 10.1016/j.vaccine.2014.05.008 24837502 10.1016/j.vaccine.2014.05.008 PMC4135535

[82] Sturgill TL, Giguere S, Berghaus LJ, Hurley DJ, Hondalus MK. Comparison of antibody and cell-mediated immune responses of foals and adult horses after vaccination with live Mycobacterium bovis BCG. Vaccine. 2014 3 10;32(12):1362–7. doi: 10.1016/j.vaccine.2014.01.032 24486362 10.1016/j.vaccine.2014.01.032

[83] Varga J, Fodor L, Rusvai M, Soos I, Makrai L. Prevention of Rhodococcus equi pneumonia of foals using two different inactivated vaccines. Vet Microbiol. 1997 6 16;56(3–4):205–12. 9226835 10.1016/s0378-1135(97)00089-8

