# Supplementary material for: Antigen-specific immunoglobulin variable region sequencing measures humoral immune response to vaccination in the equine neonate
Source: PLoS One. 2017 May 16;12(5):e0177831. doi: 10.1371/journal.pone.0177831 (PMC5433778; doi:10.1371/journal.pone.0177831)
Supplement: S1 File — Fig A. Antibody level results obtained using influenza ELISA and EIVHAI assay. (A) Antibody levels from mares in Groups C and D (n = 12) obtained using the influenza ELISA (normalized optical density, O.D.) showed agreement (r = 0.7424, p = 0.0057, Pearson correlation) with the results obtained using EIVHAI (units of antibody). (B) Pre- (median 60, minimum 8, maximum 192) and post- (72, 24, 192) vaccination EIVHAI units of antibodies were determined in serum samples from mares from Group D (n = 9) vaccinated with 2 doses of the influenza vaccine. Data points from the same mare before and after vaccination are connected by a line. There was no statistical difference (p = 0.2) between sampling times, and overall median fold-difference between before and after vaccination was 2. Fig B. Antigen-specific B cell sorting. Using a high-speed cell sorter, (A) leukocytes with size and granularity characteristic of lymphocytes were initially tested for the expression of the CD21 B cell molecule; (B) from the CD21 positive (CD21pos) population, antigen-specific B cells for KLH (KLHpos) or influenza (FLUpos) were sorted directly into individual tubes containing lysis buffer for subsequent molecular analysis. Note the rare antigen-specific cells in comparison to the overall B cell population. Fig C. Schematic of primers used for Ig amplification. A recombined Ig transcript is shown, with the IGHV gene presented in a white box. Complementarity-determining regions (CDRs) 1 and 2 are shown in thin black boxes, CDR3 spans the 3’ end of IGHV gene along with recombined D and J gene regions. The Ig heavy chain constant region is shown in a gray box. 1) To amplify and sequence IGHM transcripts, a conserved primer in the IGHV region was paired with a primer approximately 120 bases into the IGHM constant region, and resulted in products of approximately 450 bases. This primer was designed based on a conserved region among the 522 equine Ig heavy chain sequences available in GenBank (DQ1254 [file pone.0177831.s001.docx]

**Antigen-specific immunoglobulin variable region sequencing measures humoral immune response to vaccination in the equine neonate**

**Supporting information**

**Antibody level results obtained from influenza enzyme-linked immunosorbent assay and hemagglutination antibody inhibition assay**

Using pre-vaccination and post-vaccination serum samples from adult horses (mares from Groups C and D) vaccinated with 2 intramuscular doses (21-days apart) of the same equine influenza vaccine used in this study, an ELISA assay was developed in our laboratory to measure influenza-specific serum IgG levels as described in Materials and Methods (manuscript main text). Aliquots of the same samples were submitted to Cornell University Animal Health Diagnostic Center for equine influenza virus hemagglutination antibody inhibition (EIVHAI) assay, which uses the H3N8 virus. The hemagglutination inhibition antibody titer is the reciprocal of the highest dilution of serum that prevents the agglutination of red cells by influenza virus. Using Pearson correlation, antibody level results from mares from Groups C and D (n=12) obtained using the influenza ELISA (normalized optical density, O.D.) showed agreement (R=0.7424, p=0.0057) with the results obtained using EIVHAI (units of antibody) (Fig A panel A). Pre-vaccination (minus day 42) and post-vaccination (day 3 after foaling) EIVHAI units of antibodies were determined in serum samples from mares from Group D (n=9) vaccinated with 2 doses of the influenza vaccine; there was no statistical difference (p=0.2, Wilcoxon matched-pairs signed rank test) between sampling times, and overall median fold-difference between before and after vaccination was 2 (Fig A panel B).

**

**

**Fig A. Antibody level results obtained using influenza ELISA and EIVHAI assay. (A)** Antibody levels from mares in Groups C and D (n=12) obtained using the influenza ELISA (normalized optical density, O.D.) showed agreement (r=0.7424, p=0.0057, Pearson correlation) with the results obtained using EIVHAI (units of antibody). **(B)** Pre- (median 60, minimum 8, maximum 192) and post- (72, 24, 192) vaccination EIVHAI units of antibodies were determined in serum samples from mares from Group D (n=9) vaccinated with 2 doses of the influenza vaccine. Data points from the same mare before and after vaccination are connected by a line. There was no statistical difference (p=0.2) between sampling times, and overall median fold-difference between before and after vaccination was 2.

**Antigen-specific B cell sorting**

Methods for antigen-specific B cell sorting are described in the main text, and a flow cytometric dot plot figure described the cell sorting strategy (Fig B).

**
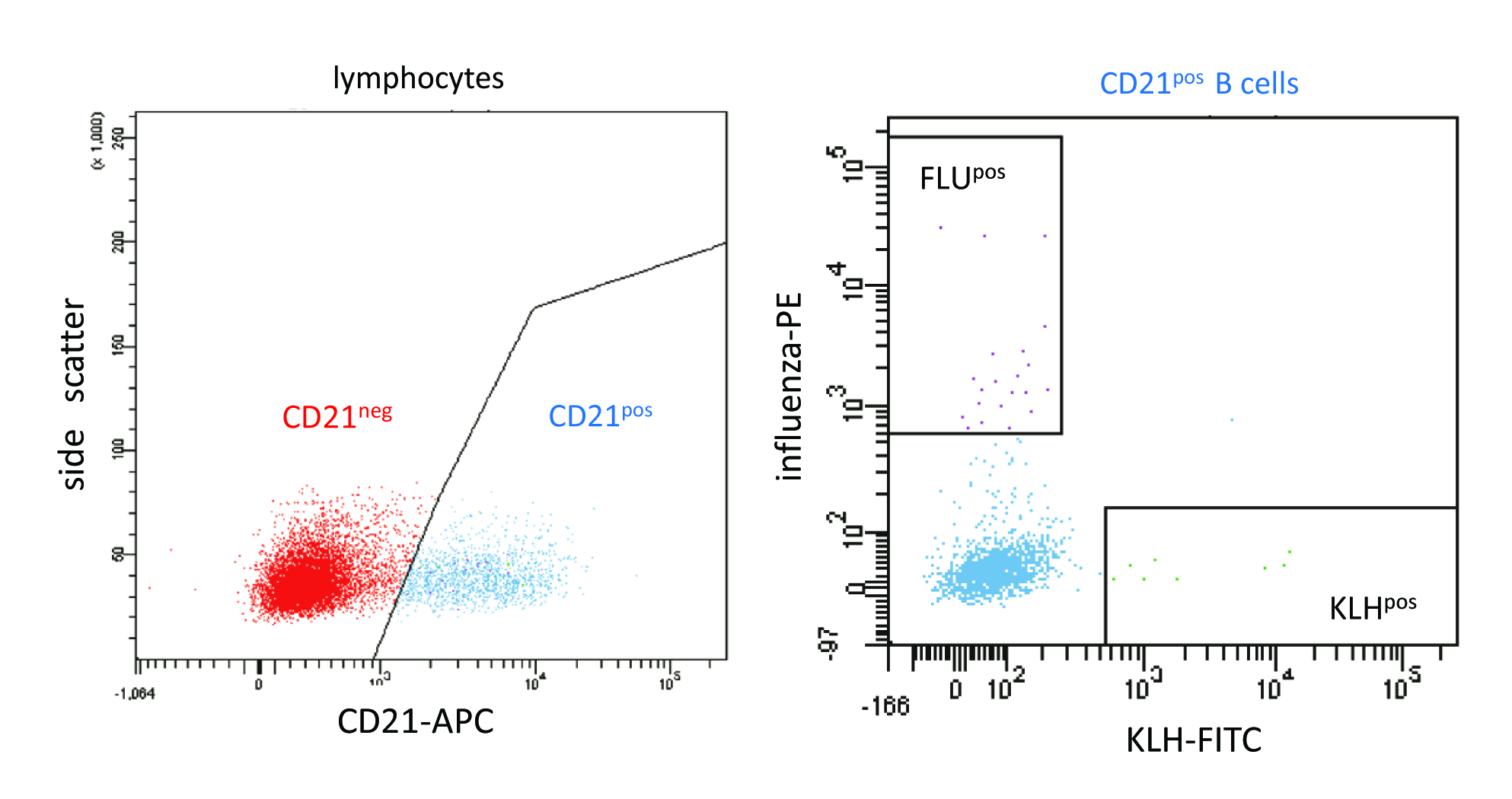
**

**Fig B. Antigen-specific B cell sorting.** Using a high-speed cell sorter, (A) leukocytes with size and granularity characteristic of lymphocytes were initially tested for the expression of the CD21 B cell molecule; (B) from the CD21 positive (CD21^pos^) population, antigen-specific B cells for KLH (KLH^pos^) or influenza (FLU^pos^) were sorted directly into individual tubes containing lysis buffer for subsequent molecular analysis. Note the rare antigen-specific cells in comparison to the overall B cell population.

**Amplification of immunoglobulin transcripts**

 Primers used to amplify antigen-specific IGHM and IGHG transcripts are shown in Fig C.

**Fig C. Schematic of primers used for Ig amplification.** A recombined Ig transcript is shown, with the IGHV gene presented in a white box. Complementarity-determining regions (CDRs) 1 and 2 are shown in thin black boxes, CDR3 spans the 3’ end of IGHV gene along with recombined D and J gene regions. The Ig heavy chain constant region is shown in a gray box. 1) To amplify and sequence IGHM transcripts, a conserved primer in the IGHV region was paired with a primer approximately 120 bases into the IGHM constant region, and resulted in products of approximately 450 bases. This primer was designed based on a conserved region among the 522 equine Ig heavy chain sequences available in GenBank (DQ125413-DQ125458, HM175886-HM176092, HQ403608-HQ403643, KC549680-KC549800, KF748698-KF748792, KJ741369-KJ741385), the equine genome sequence IGHV region (NW_001876796), and other projects in our laboratory (50). When no IGHV-IGHG product was detected, other conserved IGHV forward primers (5’ GTGGTTCTTCCTCTTTCTGGTG 3’ and 5’ GCTCCTACATGTGTCCTGTCC 3’) were used in regular and semi-nested RT-PCR but no additional PCR products were obtained. 2) To amplify the IGHG constant region, primers conserved among the 7 IGHG isotypes were used and produced products of 71 to 74 bases. 3) To sequence the variable region of IGHG transcripts, the same conserved primer in the IGHV region used in set 1 was now paired with a reverse primer in the IGHG constant region that was conserved among the 7 IGHG isotypes. IGHV-IGHG products ranged from approximately 550 to 600 bases.

**Antigen-specific IGHM CDR3 length distribution**

The CDR3, the most hypervariable region of the Ig, is used to characterize the Ig repertoire variation over time. The CDR3 length distribution was assessed from KLH-specific IGHM sequences as described previously (4). For statistical analysis, the Shapiro–Wilk normality test revealed that, in most cases, data did not fit a normal distribution and the Wilcoxon–Mann–Whitney Rank Sum test with Bonferroni correction for multiple comparisons was used (GraphPad Software, San Diego, CA). For keyhole limpet hemocyanin (KLH)-specific IGHM (Fig C), both vaccinated groups (Groups A and B) and non-vaccinated group (Group E) showed a majority of sequences harboring CDR3 lengths of 10 to 20 amino acids over time (Fig D). No statistical differences (p>0.05) in CDR3 length were found within a group over time or between groups on the same day. For influenza-specific IGHM, a majority of CDR3 lengths of 12 to 22 amino acids were identified in all groups (Groups C, D and E) (Fig E). No statistical difference (p>0.05) in CDR3 length was found between groups.

**
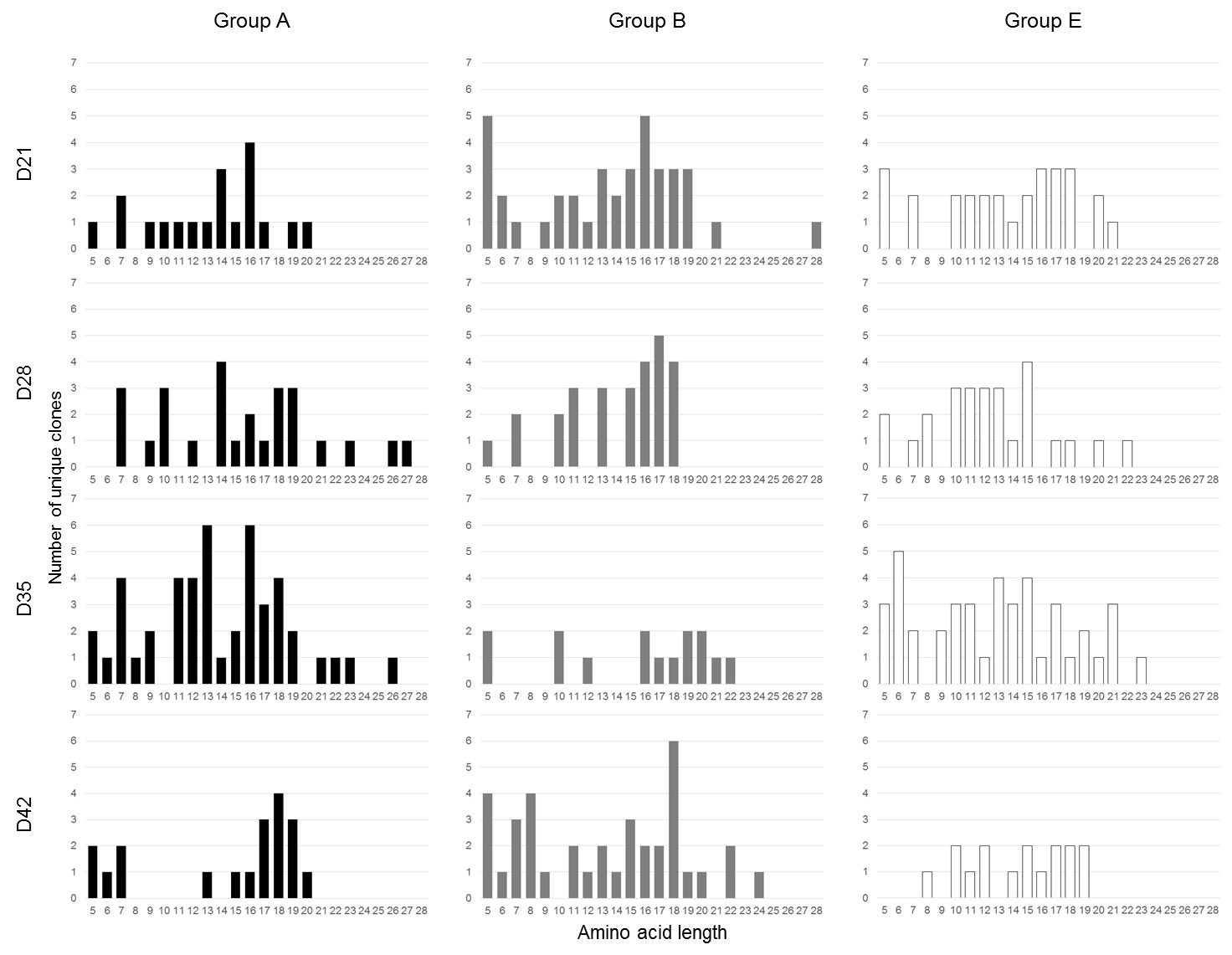
**

**Fig D. KLH-specific IGHM CDR3 length distribution.** The CDR3 amino acid sequence length of KLH-specific IGHM transcripts from foal samples is presented on the x-axis, and the number of unique clones on the y-axis. Data are shown by sample time (age) for each group (Groups A, B and E).

**
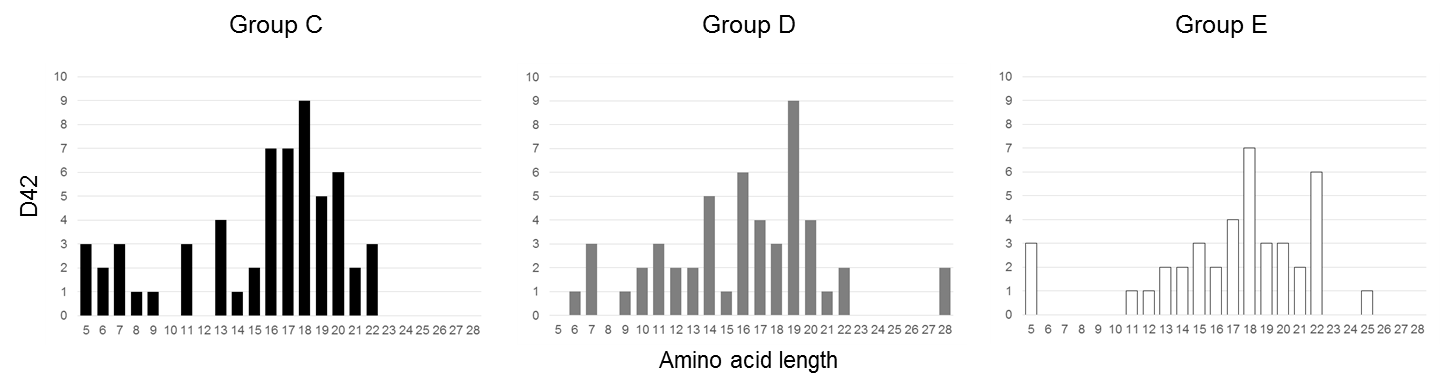
**

**Fig E. Influenza-specific IGHM CDR3 length distribution.** The CDR3 amino acid sequence length of influenza-specific IGHM transcripts from foal samples is presented on the x-axis, and number of unique clones on the y-axis. Data are shown for sample time day 42 for each group (Groups C, D and E).

**Leukocyte cytokine production after *in vitro* stimulation with KLH or influenza proteins**

Approximately 5x10^6^ peripheral blood mononuclear cells preserved frozen in medium from days 3 and 42 of each foal group were thawed as described in Materials and Methods of the manuscript main text. Cells were resuspended in 1ml RPMI medium containing L-glutamine plus 10% fetal bovine serum and 1X antimycotic/antibiotic (Gibco Life Technologies, Thermo Fisher Scientific, Grand Island, NY). After a washing step, cells were counted and evaluated for cell viability using Trypan blue exclusion. One million viable cells in 1ml medium were not-stimulated (baseline control) or stimulated with10 ug/ml KLH (Sigma-Aldrich Co., St. Louis, MO), 2 ug/ml equine influenza N8 neuraminidase protein (BEI Resources, NIAID, NIH), or 5 ug/ml phytohemagglutinin (PHA, Sigma-Aldrich Co.) for 60 hours at 37^o^ C, 5% CO_2_. Brefeldin-A (Sigma-Aldrich Co.) was added at 10 ug/ml in the last 4 hours of culture. Cells were harvested, washed with phosphate buffered solution (PBS), and resuspended in 200 uL PBS. To measure cytokine production upon stimulation, cell surface was initially labeled with monoclonal antibody fluorescein (FITC)-conjugated mouse anti-horse CD4 (clone MCA1078F, AbD Serotec, Bio-Rad Laboratories, Hercules, CA) for 45 min. Cells were washed 3 times in PBS, and fixed (Cytofix/CytoPerm^TM^, BD Biosciences, San Jose, CA) for 20min. After 2 washes with a cell membrane permeabilizing solution (Cytofix/CytoPerm^TM^, BD Biosciences), intracellular staining was performed using monoclonal-antibody phycoerythrin (PE)-conjugated IL-4 (clone MCA1820PE, AbD Serotec, Bio-Rad Laboratories), and monoclonal antibody Alexa fluor® 647-conjugated interferon-gamma (IFNg) (clone MCA1783A647, AbD Serotec, Bio-Rad Laboratories) for 45 min. After 3 washes with the permeabilizing solution, cells were fixed in 200uL 2% paraformaldehyde solution in PBS. Three-color flow cytometry was performed to measure the percent of CD4 T cells expressing IL-4 or IFNg using a BD FACSCalibur^TM^ equipped with argon and red diode lasers (BD Biosciences). A minimum of 10,000 events were collected in a gate determined based on lymphocyte scattering characteristics (size, forward scatter; granularity, side scatter), and analyzed with BD CellQuest Pro Software (BD Biosciences). Negative controls confirmed that non-specific staining was less than 2% of lymphocyte-gated cells.

Data (percent positive cells) were collected for total leukocyte or CD4+ T cell expressing either cytokine, since many foals showed a bright and a dim population of CD4+ cells. Percent values for stimulated leukocytes were normalized to (divided by) values measured for the respective non-stimulated (baseline) samples for day 3 and day 42, and values are shown as relative expression above baseline. Data distribution was not normal according to Shapiro-Wilk test. Median values for cytokine (IL-4 or IFNg) positive leukocytes were compared between day 3 and day 42 for each group or between groups using Wilcoxin-signed rank test; Type I error was set at 5%. Groups included: vaccinated foals from non-vaccinated mares (KLH, Group A; influenza, Group C); vaccinated foals from vaccinated mares (KLH, Group B; influenza, Group D); and non-vaccinated foals from non-vaccinated mares (Group E). Data analysis revealed no statistical differences (p>0.05) between days 3 and 42, or between groups when cells were stimulated with the immunogens (Fig F). Similar results were obtained when cytokine data was collected for CD4+ T cells (i.e. CD4+IL4+ cells or CD4+IFNg+ cells). Analysis of cytokine production by comparison of mean fluorescence intensity (MFI) also resulted in no difference between groups (p>0.05).

**Fig F. Cytokine production by foal peripheral blood leukocytes stimulated *in vitro* with KLH or influenza protein.** Peripheral blood isolated leukocytes from days 3 (black bars) and 42 (white bars) were not-stimulated (baseline) or stimulated *in vitro* with KLH, influenza or PHA (positive control). After 60hrs in culture, cells were labeled for the expression of IL-4 and IFNg. Percent values of IL-4-positive or IFNg-positive stimulated cells were normalized to (divided by) values measured for the respective non-stimulated samples. Data are shown as relative expression above baseline, and by group: vaccinated foals from non-vaccinated mares (KLH, Group A; influenza, Group C); vaccinated foals from vaccinated mares (KLH, Group B; influenza, Group D); and non-vaccinated foals from non-vaccinated mares (Group E). For PHA-stimulated cells, data are shown for Groups A, B and E. There were no statistical differences (p>0.05) between days 3 and 42, or between groups.
